# Practical quantum-enhanced receivers for classical communication

**DOI:** 10.1116/5.0036959

**Published:** 2021

**Authors:** I. A. Burenkov, M. V. Jabir, S. V. Polyakov

**Affiliations:** 1 National Institute of Standards and Technology, Gaithersburg, Maryland 20899, USA; 2 Joint Quantum Institute and University of Maryland, College Park, Maryland 20742, USA; 3 Department of Physics, University of Maryland, College Park, Maryland 20742, USA

## Abstract

Communication is an integral part of human life. Today, optical pulses are the preferred information carriers for long-distance communication. The exponential growth in data leads to a “capacity crunch” in the underlying physical systems. One of the possible methods to deter the exponential growth of physical resources for communication is to use quantum, rather than classical measurement at the receiver. Quantum measurement improves the energy efficiency of optical communication protocols by enabling discrimination of optical coherent states with the discrimination error rate below the shot-noise limit. In this review article, the authors focus on quantum receivers that can be practically implemented at the current state of technology, first and foremost displacement-based receivers. The authors present the experimentalist view on the progress in quantum-enhanced receivers and discuss their potential.

## I. INTRODUCTION

The communication capacity crunch is upon us,^1,2^ owing to the exponential expansion of the Internet. With monthly Internet traffic of 200 exabytes at the time of writing, the underlying communications systems will no longer be able to support the service reliability and Internet traffic congestion will only worsen as the exponential trend continues. Claude Shannon analyzed and described the limits of a communication channel.^3^ He found a universal relation between information capacity, available channel resources, and noise. The connection between information and physics turns out to be even more fundamental. This connection is now well-established as a result of the progress in information theory and computer science, on one hand,^4,5^ and quantum physics on the other.^6^ With physical measurement at the heart of communication, the fundamental communication channel limits are related to fundamental properties of measurements.

Quantum theory established tools that quantitatively connect physical measurement and communication. In 1962, Gordon found the maximal capacity of electromagnetic (bosonic) channels from first principles.^7^ The connection between the channel’s physical resource and its capacity was solidified in the subsequent work.^8–15^ On one hand,^9,10^ there is a fundamental limit of measurement accuracy that leads to occasional errors in discriminating between physical states used for communication called Helstrom bound (HB). On the other hand, it was found that commonly used detection techniques such as homodyne/heterodyne measurement even with the ideal components are limited by measurement noise^7,11–15^ due to the quantum nature of the photoelectric effect and the Poisson photon statistics of the coherent light.^16^ This inherent noise is called shot noise. This noise prevents reaching the rate of errors prescribed by Helstrom’s work.

Thus, at least potentially, quantum measurements can surpass the capabilities of classical measurements and improve channel capacity (to within Gordon’s bound). A new field of research was born. The main goal of this research is to use quantum effects to surpass the shot-noise limited measurement. First practically attainable quantum measurement-based receivers were proposed in 1970s.^17,18^ This pioneering work was followed by further theoretical and experimental research, for instance.^19–26^ The field became particularly active in the late 2010s as highly efficient, low dark noise single-photon detectors became available.^27^

Although there are reviews on discrete quantum state discrimination,^28–33^ there is no comprehensive review of experimental efforts in state discrimination of the continuous-variable states. In this review, we focus on the state-of-the art quantum measurement schemes and communication protocols for classical communications with finite sets of continuous-variable states, such as coherent states. Because we primarily describe receivers that have been practically implemented, the central attention is devoted to coherent displacement-based receiver designs. Averting the capacity crunch in global communications may require paradigm-shifting research and engineering efforts. Quantum measurement could provide new tools that will help take full advantage of communication channels—up to the theoretical maximum—and thus enable this paradigm shift.

This review is organized as follows. In Sec. II, we briefly review the theoretical foundations of classical and quantum-enabled channels. We introduce a simple classification of the communication channels based on the type of encoding and the type of measurement. In Sec. III, we discuss conventional and novel communication protocols used for coherent optical communication. We compare power limited and bandwidth limited encodings and the trade-off between their resource efficiencies. In Sec. IV, we discuss displacement-based quantum receivers for discrimination of coherent states. The two main classes of the receivers are considered: receivers with adaptive displacement and passive displacement receivers. In Sec. V, we outline research efforts beyond displacement receivers and beyond the use of coherent states in noiseless communication channels. Section VI summarizes advantages and challenges of the potential widespread use of quantum measurement for communication and concludes the review.

## II. THEORY OF DETECTION; CLASSICAL SHOT-NOISE LIMIT AND QUANTUM HELSTROM BOUND

### A. Quantum-enabled channels

Quantum theory revisited the fundamentals of communication. The calculation of information capacity limits of conventional communication channels from the first principles became possible. Then, questions on using quantum enhancement to improve conventional communication channels emerged. Although we expect a higher capacity for a quantum-enabled channel, additional steps may be required to take advantage of it. A communication link requires an encoding scheme that maps user information to physical states and a measurement device for a physical state detection. Thus, on the most practical level, quantum properties of physical states and measurement need to be considered, potentially limiting the practically accessible channel capacity. We will start from the most abstract analysis and then consider practical constraints.

In general, a quantum enabled channel supports (1) classical encoding and measurement, (2) classical encoding and quantum measurement, (3) quantum encoding and classical measurement, and (4) quantum encoding and measurement. The information capacity of quantum-enabled channels is bounded from above by Holevo’s theorem.^8^

From a quantum standpoint, electromagnetic waves are described by expanding them to a series of orthogonal modes and prescribing each mode a discrete number of excitations, i.e., photons. For the sake of simplicity, we assume communication via a single spatial mode, which is most commonly the case. Then, the number of orthogonal modes is directly related to the frequency bandwidth *B* of the channel. The average number of photons per state *n* is directly proportional to the average energy: *E* = *ħωn*. The average power is *W* = *EB* = *nħωB*. After substituting the maximal achievable entropy per optical mode to the Holevo theorem, one finds the capacity of a lossless and noiseless quantum-enabled channel:^7^

(1) $$C_{\text{Q}}=B\;\log_{2}\left(1+\frac{W}{\hbar \omega B}\right)+\frac{W}{\hbar \omega }\log_{2}\left(1+\frac{\hbar \omega B}{W}\right).$$

Therefore, the number of modes and the average energy of an optical state fully describe the physical resource use when electromagnetic waves are used as information carriers. This important result is referred to as Gordon capacity (or Holevo bound). To aid comparison, the channel capacity is often divided by the channel bandwidth. Then, normalized channel capacity *C*_Q_/*B* conveniently characterizes the spectral efficiency. Formally, the spectral efficiency is measured in bits, but often units bits/s/Hz are used to emphasize the physical meaning of *C*/*B* as a measure of data rate in bits per second over a channel with a bandwidth of 1 Hz. *C*_Q_/*B* is also used for classification of communication protocols^34,35^

(2) $$C_{\text{Q}}/B=\log_{2}\left(1+\frac{W}{\hbar \omega B}\right)+\frac{W}{\hbar \omega B}\log_{2}\left(1+\frac{\hbar \omega B}{W}\right).$$

In a classical limit, *W* ≫ *Bħω*, so the second term nearly vanishes, and the capacity becomes *C*_Q_ ≈ *C*_Shannon_ = *B* log_2_(1 + *W*/(*ħωB*)). This result is identical to a classical channel capacity given by the Shannon limit, where *W*/(*ħωB*) is a signal to noise ratio.

**FIG. 1.**
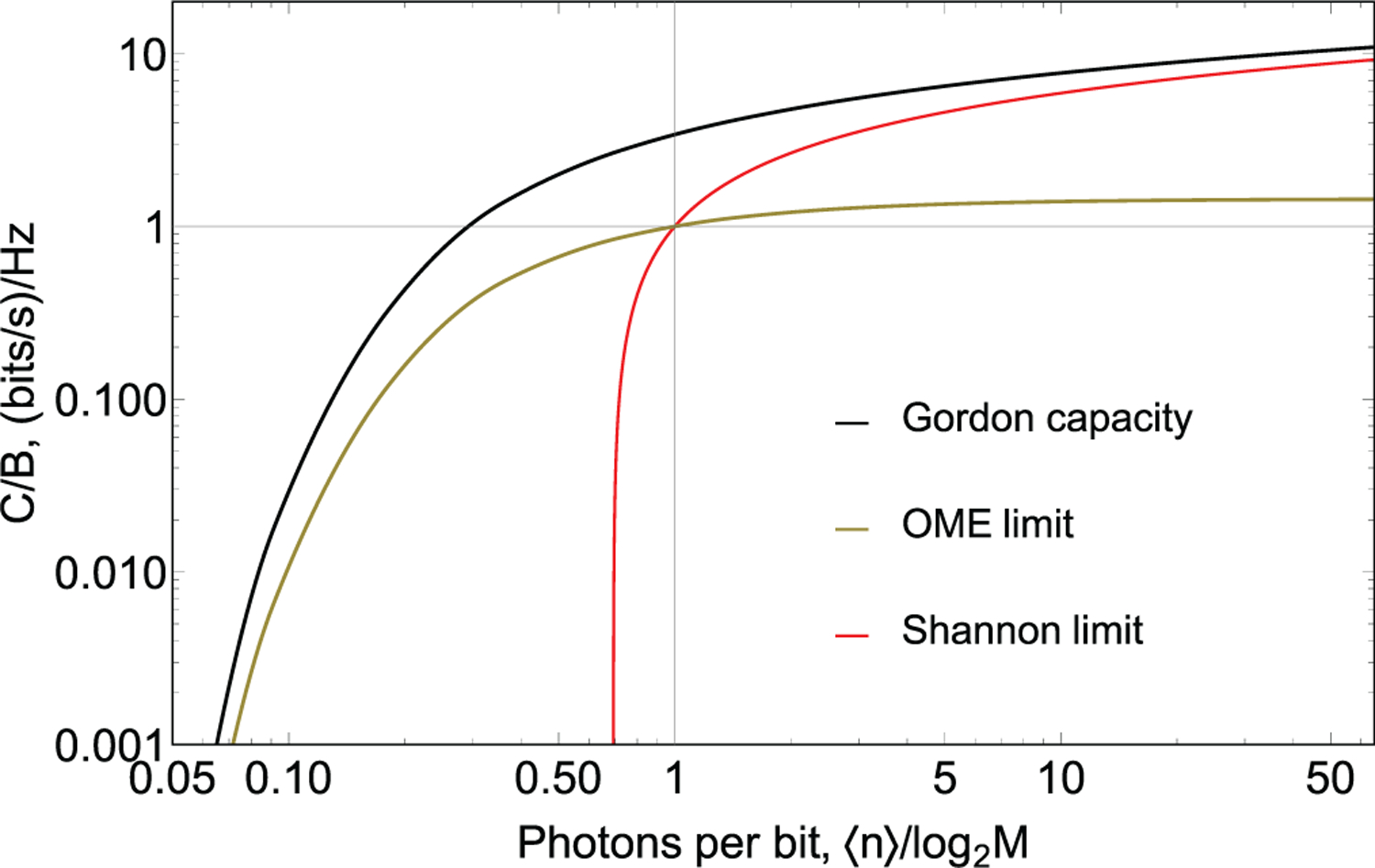
Classical and quantum limits to channel capacity

In a photon-starving regime, *W* ≪ *Bħω* capacity is mainly defined by the second term in (2). This result can be interpreted as follows. For low input power, one can use several orthogonal modes and, each time they send the entire available energy (a single photon) in one mode. The more modes are available, the more bits of information can be encoded per photon. For example, if one photon can be sent per time interval *T*, then for an available bandwidth *B*, one can divide this interval into *M* = *BT* slots. The information can be encoded by sending a photon in a particular time slot. The number of encoded bits is log_2_*BT*. Therefore,

(3) $$C_{\text{OME}}=\frac{\log_{2}BT}{T}=\frac{W}{\hbar \omega B}\log_{2}\left(1+\frac{\hbar \omega B}{W}\right),$$

where OME stands for orthogonal mode encoding. Spectral efficiencies *C*_Q_/*B*, *C*_Shannon_/*B* and *C*_OME_/*B* are shown as a function of energy efficiency defined as average number of photons used to transmit 1 bit of information in Fig. 1.

Note that this simple result is based on the assumption of a noiseless and lossless channel. In this ideal case only encoding using Fock states in conjunction with ideal photon-number resolving (PNR) measurement can attain the Gordon capacity, Table I. Typically, optical channels exhibit a significant loss. The upper bound (2) can be corrected by changing assumptions. In particular, adding a model for losses leads to a different capacity bound.^36–38^ Any practical optical communication system requires physical states that are resilient to optical loss, at least to some extent. To this end, classical states, especially coherent states of light, are particularly useful. In this review, we focus on channels with classical encoding and quantum measurement.

To design a practical digital communication system, an encoding method to map digital information on transmitted physical states is needed. The set of states {|*Ψj*〉} is called an alphabet; it can be of an arbitrary length *M*. We assume equiprobable states and a noiseless channel, Table I. How well can the alphabet symbols be distinguished? To answer this question quantitatively, we use symbol error rate (SER), the probability that a transmitted symbol is received incorrectly, *P*. Helstrom determined that the lower bound on this error is related to an overlap of the alphabet states.^10^ One uses the square root measure (SRM) method^8,23,39,40^ to find the Helstrom bound (HB). This method relies on a Gram matrix defined as

(4) $$G_{mj}=\langle \psi_{m}|\psi_{j}\rangle .$$

Note that the dot product in (4) cannot be zero for coherent states. Indeed, in Fock basis, one writes

(5) $$|\psi_{j}\rangle =e^{-\frac{|x{|}^{2}}{2}}\left(|{\mathrm{vac}}_{j}\rangle +\alpha |1_{j}\rangle +\frac{1}{2}\alpha^{2}|2_{j}\rangle +\cdots \right),$$

where *α* is a coherent state parameter. Recall that 〈vac_*m*_|vac_*j*_〉 = 1 even if modes *m* and *j* are orthogonal. This property of coherent states is important for other applications such as quantum fingerprinting.^41^ Interestingly, even if the communication alphabet uses quantum states with no vacuum component, any loss in a channel admixes the vacuum component to the initial state. The error probability bound for a quantum receiver can be written as the square root of Gram matrix elements

(6) $$P_{\text{HB}}=1-\sum_{m=1}^{M}{\left|{\left(G^{1/2}\right)}_{mm}\right|}^{2}/M.$$

In general, Helstrom bound cannot be found analytically. For some encodings, $G^{1/2}$ has an analytical form. We will give examples of Helstrom bounds for typical encodings in Sec. III.

Because Holevo theorem bounds channel capacity and HB puts a limit on error rate, the two bounds cannot be directly compared. However, HB gives the resource use, i.e., the required power and bandwidth to reach a certain error probability. Thus, to benchmark an encoding, the error probability is fixed. Then the normalized data rate *R*/*B* and the required power are compared to the normalized channel capacity *C*(*W*)/*B*. Obviously, for sufficiently low SER $P_{\text{HB}}^{\prime }$ and a lossy communication channel, *R*_HB_/*B* < *C*(*W´)*/*B*, where *W´* is the power required to achieve the SER $P_{\text{HB}}^{\prime }$. It is very important to note here that HB merely establishes the lowest possible error probability, but does not guarantee a measurement method capable of achieving the HB. Hence, we expect that experimental spectral efficiency *R*_E_/*B* ≤ *R*_HB_/*B* < *C*(*P*)/*B*.

### B. Classical channels

Unless OME is used, classically, the information capacity is given by the Shannon theorem^3^

$$C_{\text{Shannon}\;}=B\log_{2}\left(1+\frac{W}{N}\right).$$

This classical model does not specify the origin of channel noise *N*. Naively, this noise is a property of a communication channel and can be arbitrary small, which would result in the infinite channel capacity. In reality, noise is a fundamental property of any measurement. Because communication cannot occur without a physical measurement at the receiver, it is the measurement noise that would limit an otherwise noiseless channel. Although noise can be introduced *ad hoc* into a classical model of measurement, it is much more convenient to derive the minimal measurement noise using a quantum mechanical description of an otherwise classical measurement.^7,42,43^ A typical classical receiver measures the optical signal via heterodyne and/or homodyne measurements. In both cases, the signal undergoes interference on a beam splitter with a local oscillator (LO). The LO is a coherent state with the same optical frequency as the signal carrier in the case of the homodyne and a different frequency in the case of the heterodyne. After interference, the signal is detected on one or more detector(s). In all cases, there will be a current at the detector, and hence there will be shot noise. Assuming the detection efficiency of unity, the information capacity of coherent homodyne and heterodyne receivers is^7^

(7) $$\begin{array}{l} \;\;C_{\text{heterodyne}\;}=B\log_{2}\left(1+\frac{W}{\hbar \omega B}\right),\\ C_{\text{homodyne}\;}=\frac{B}{2}\log_{2}\left(1+4\frac{W}{\hbar \omega B}\right). \end{array}$$

We see that the measurement-induced noise is proportional to the channel’s bandwidth, and the dependence of capacity on power and bandwidth in (7) is similar to the first term of the Gordon capacity, cf. Eq. (1).

For OME, a so-called direct detection measurement can be used. In principle, orthogonal optical modes can be physically separated without introducing extra noise or loss. Once separated, each mode can be separately measured with a detector. For instance, if spectral modes were used, a dispersive element such as a grating could be employed, followed by *M* spatially separated detectors. For pulse position multiplexing (PPM), when a position of a short pulse within a larger temporal window encodes information, one time-resolving detector is sufficient because modes are separated in time. A successful detection occurs when light is detected in one and only one mode. Although the exact analytical expression for the OME capacity in a classical Poisson channel is not known,^44,45^ channel capacity scales like $\frac{W}{\hbar \omega B}\log_{2}\left(1+\frac{\hbar \omega B}{W}\right)$ in a limit of weak optical input.^46^ This limit is identical to the second term of the Gordon capacity, cf. (3).

We see that *C*_Q_ > *C*_heterodyne_, *C*_homodyne_, *C*_OME_. Therefore, channel capacity of the quantum-enabled channel exceeds that of the classical channel. In derivations above, one does not specify a modulation scheme to obtain channel capacity. Finding the upper bound for SER requires selecting a modulation scheme. The uncertainty due to shot noise on the detector^12,47^ leads to state discrimination errors. The lowest classically attainable symbol error rate is often referred to as shot noise limit (SNL), quantum noise limit (QNL), or standard quantum limit (SQL). We will give examples of SNL derivations for particular modulation protocols in Sec. III.

To benchmark an encoding, a SER *P* is fixed (at a sufficiently low value). Then the normalized data rate *R*/*B* and the required power can be compared to the normalized channel capacity *C*(*W*)/*B*. Thus, the highest attainable data rates for classical and quantum-enabled channels as well as Holevo bound and Shannon limit can be presented on the same graph. As we will see below, *R*_SNL_(*W´*)/*B* < *R*_HB_(*W´*)/*B* < *C*_Q_(*W´*)/*B*, where *W´* is a fixed power. Note that because we explicitly assume the measurement method to compute SNL, the classical lowest possible error probability *P*_SNL_ can in principle be achieved using ideal components, as opposed to *P*_HB_, because the ideal quantum measurement method might be unknown.

Table I summarizes channel capacity and SER bounds and the assumptions that are required to derive them.

## III. CONVENTIONAL and NOVEL COMMUNICATION PROTOCOLS

**FIG. 2.**
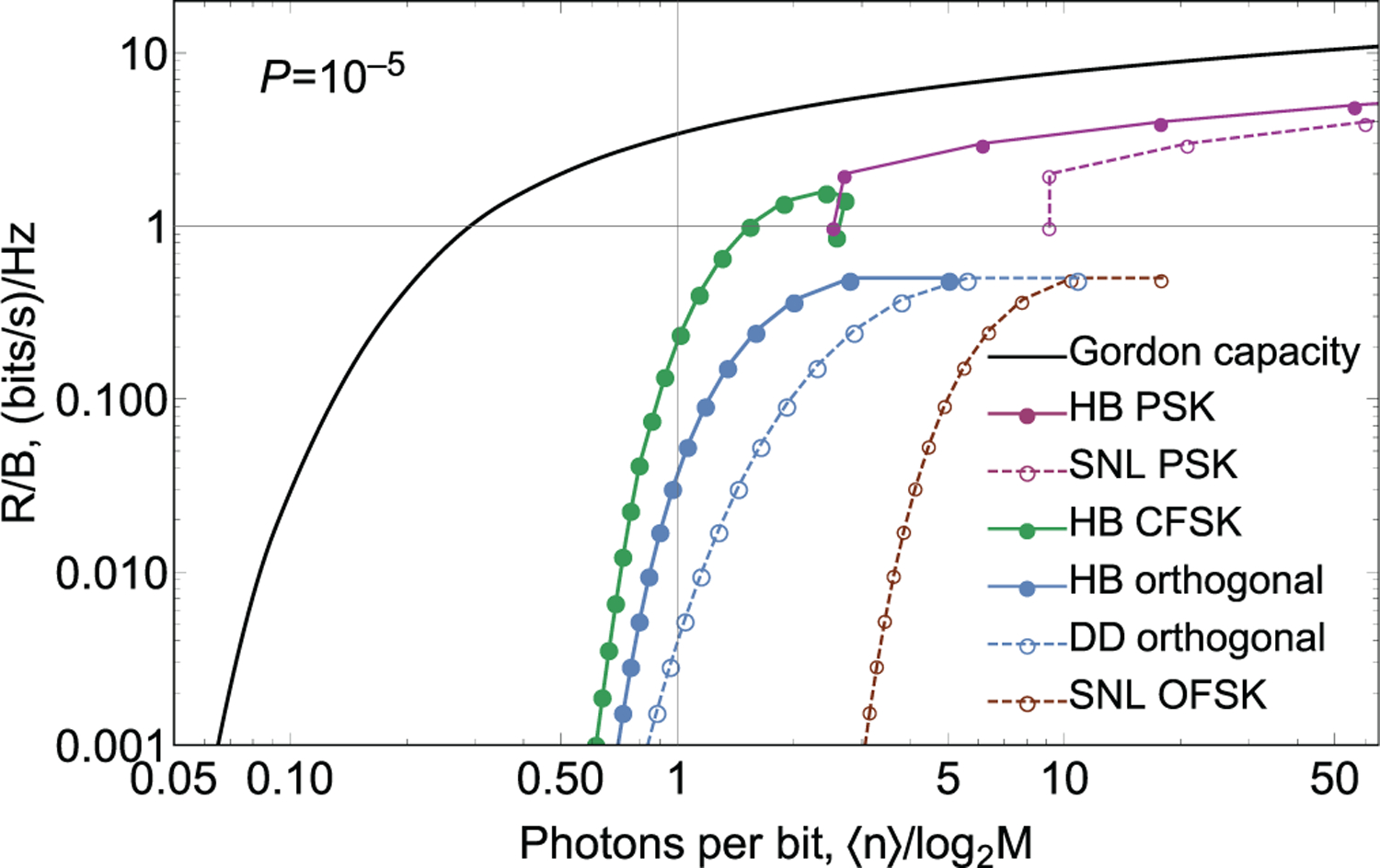
Resource use per bit for different communication protocols. Bandwidth and the theoretical minimum energy per bit requirements are shown for classical, quantum state discrimination of some communication protocols assuming a symbol error rate *P* = 10^−5^. The protocols with the same modulation method, but different alphabet lengths *M* are connected with colored lines. Power-limited protocols are above *R*/*B* = 1 line and bandwidth-limited protocols are below *R*/*B* = 1 line.

In digital communications, the ratio between data rate and bandwidth *R*/*B* gives the spectral efficiency of the communication protocols. Two main families of modulation schemes are generally distinguished: power-limited *R*/*B* > 1 and bandwidth-limited *R*/*B* < 1, Fig. 2. The power-limited family includes such encodings as pulse amplitude modulation (PAM), quadrature amplitude modulation (QAM), phase-shift keying (PSK), and others. In these modulation schemes, the bit rate *R* for a fixed signal pulse duration grows as log_2_*M* as the number of states in the alphabet *M* increases. Communication bandwidth *B* remains constant, which means that the spectral efficiency *R*/*B* improves with *M*. However, power-limited modulation schemes using longer communication alphabets *M* require more power than these with shorter alphabets for reliable communication because it is generally harder to discriminate a larger number of non-orthogonal states. The maximal possible *R*/*B* is set by the power limit of the communication channel. While energy per symbol requirements increase as a power function of *M*, the number of encoded bits increases logarithmically. Thus, even though spectral efficiency *R*/*B* improves, energy requirements per bit increase exponentially.

The bandwidth-limited *R*/*B* < 1 family includes pulse position modulation (PPM), biorthogonal and simplex signal modulation, and orthogonal frequency-shift keying (OFSK). These encodings typically use classically orthogonal states. The number of bits carried with each signal pulse depend on the alphabet length as log_2_*M*, the same dependence as for power-limited protocols. However, the bandwidth occupied by orthogonal communication symbols grows linearly with the alphabet length *M*. The energy efficiency improves with *M* because each signal pulse carries more information, while the energy required for reliable discrimination does not depend on the number of orthogonal signals. The spectral efficiency *R*/*B* of bandwidth-limited protocols decreases as *M*/(log_2_*M*), Fig. 2. The largest *M* is given by the bandwidth limit of the communication channel.

We will discuss different modulation methods, review their theoretical limits for detection error rates, and compare their performance with the fundamental channel capacity. We will focus on encoding schemes that have been actively considered for quantum-enabled communication experiments.

### A. Binary protocols

Binary protocols are well studied, and they are a rare case where analytical expressions for error rate limits can be found, see Table II. It is not surprising that the first quantum receiver outperforming SNL was proposed for the binary modulation.^17^ In addition, the first projection measurement that achieves the HB (or the optimal projection) was found for binary encodings.^10,18^ Here we discuss binary encodings based on amplitude and phase modulations.

The binary PSK (BPSK) uses two coherent states with opposite phases for encoding and encodes exactly one bit per symbol

$$s_{0}=|-\alpha \rangle ,$$

$$s_{1}=|\alpha \rangle .$$

**FIG. 3.**
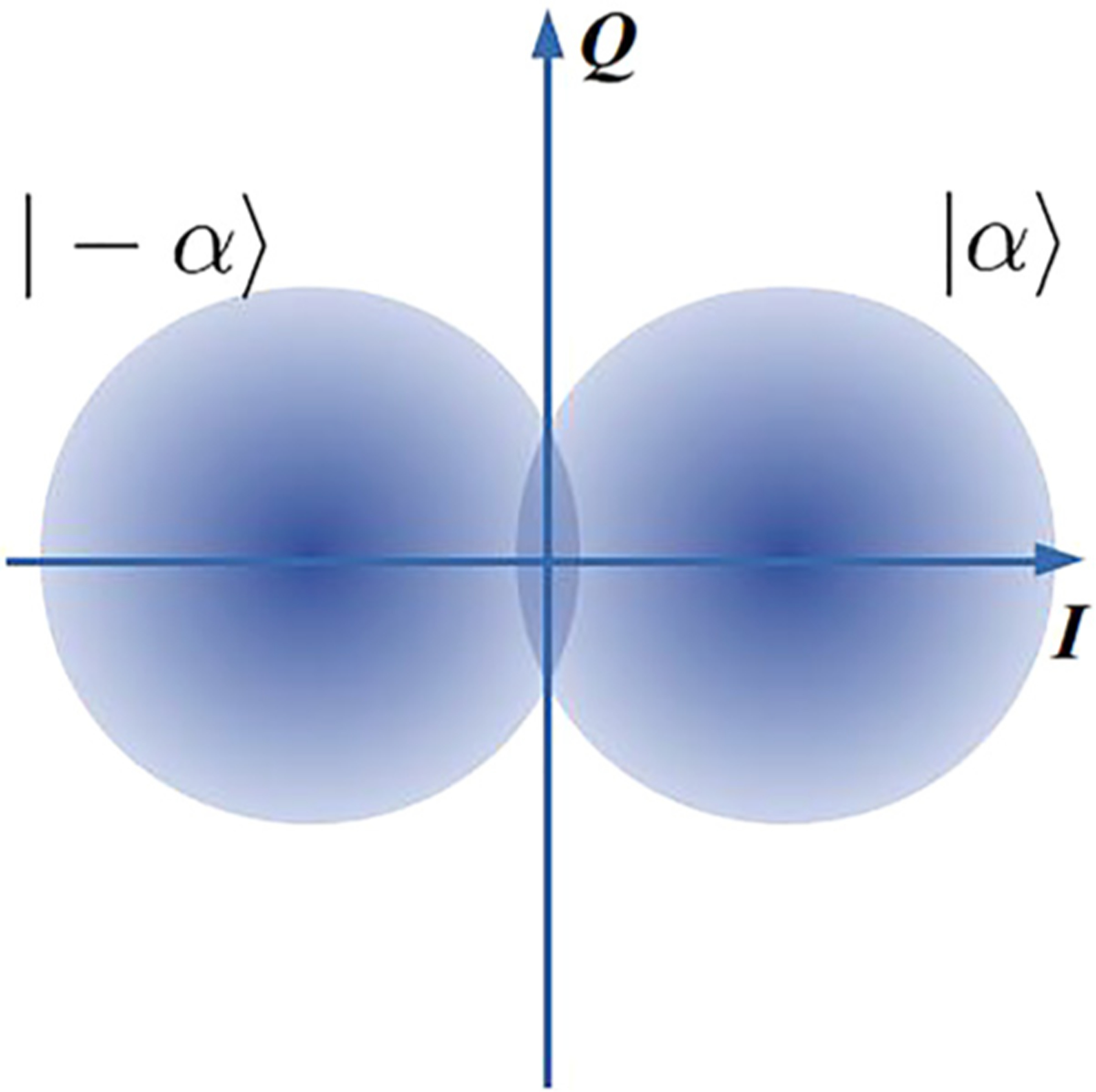
Binary phase shift keying constellation diagram.

The corresponding constellation diagram is shown in Fig. 3. Fuzzy circles represent coherent states on a phase diagram, a distance from the state to the origin is proportional to the square root of the average number of photons in the state, and an average phase is measured as the angle between the positive direction of axis *I* and the vector from the origin to the center of the coherent state. Because both BPSK symbols *s*_0_ and *s*_1_ are states of the same optical mode, this encoding is non-orthogonal even if one can neglect the vacuum component, cf. (5). Faint states *s* can significantly overlap. The optimal classical discrimination of the BPSK signals can be achieved via a homodyne measurement. The only relevant measurement value for BPSK is the projection of the measured state on the in-phase quadrature (*I* axis in Fig. 3). The probability density function to receive a projection *x* when state *s*_*i*_ was sent is

(8) $$p\left(x|s_{i}\right)=\frac{1}{\pi }\exp\left(-{\left(x-\sqrt{2}ℜ\left[s_{i}\right]\right)}^{2}\right)=\frac{1}{\pi }\exp\left(-{\left(x\pm \sqrt{2n}\right)}^{2}\right),$$

where *n* = 〈*n*〉 = |*α*|^2^ is the average number of photons in a state *s*_*i*_. A decision that the input state is *s*_0_ is made if the measured *x* < 0; otherwise, if *x* > 0, the decision is *s*_1_. Therefore, to find the probability of a discrimination error, we need to compute the probability of measuring *x* > 0 when *s*_0_ was sent (or the probability of measuring *x* < 0 when *s*_1_ was sent),

(9) $$P_{\text{SNL}}^{\text{PSK}}=\int_{0}^{\infty }p\left(x|s_{0}\right)dx=\frac{1}{2}(1-\mathrm{erf}(\sqrt{2n})).$$

The HB can be readily found as

(10) $$P_{\text{HB}}^{\text{PSK}}=\frac{1}{2}\left(1-\sqrt{1-e^{-4n}}\right).$$

As we expect, *P*_HB_ < *P*_SNL_.

The BPSK constellation is similar to a binary on-off keying (OOK) (i.e., *s*_0_ = 0, *s*_1_ = |*α*〉) when the origin is shifted to the center of the left state in Fig 3. Thus, we immediately get^20^

(11) $$\begin{array}{l} \;\;\;\;\;P_{\text{SNL}}^{\text{OOK}}=\frac{1}{2}\left(1-e^{-n}\right),\\ P_{\text{HB}}^{\text{OOK}}=\frac{1}{2}\left(1-\sqrt{1-e^{-n}}\right). \end{array}$$

Note that the classical measurement of OOK states distinguishes coherent states from vacuum states, and this measurement does not require a heterodyne; $P_{\text{SNL}}^{\text{OOK}}$ is based on direct optical power measurement (direct detection). OOK requires four times higher peak energy and two times higher average signal energy than BPSK to match its quantum discrimination error rate bound, HB. This inefficiency can be explained by calculating the geometrical distance between signal vectors $d_{01}^{\text{PSK}}=2\alpha$ and $d_{01}^{\text{OOK}}=\alpha$.^34^

Binary protocols can carry only one bit of information with each signal pulse. It may be beneficial to encode more than one bit of information per signal pulse, i.e., by using larger encoding alphabets.

### B. *M*-ary PSK

**FIG. 4.**
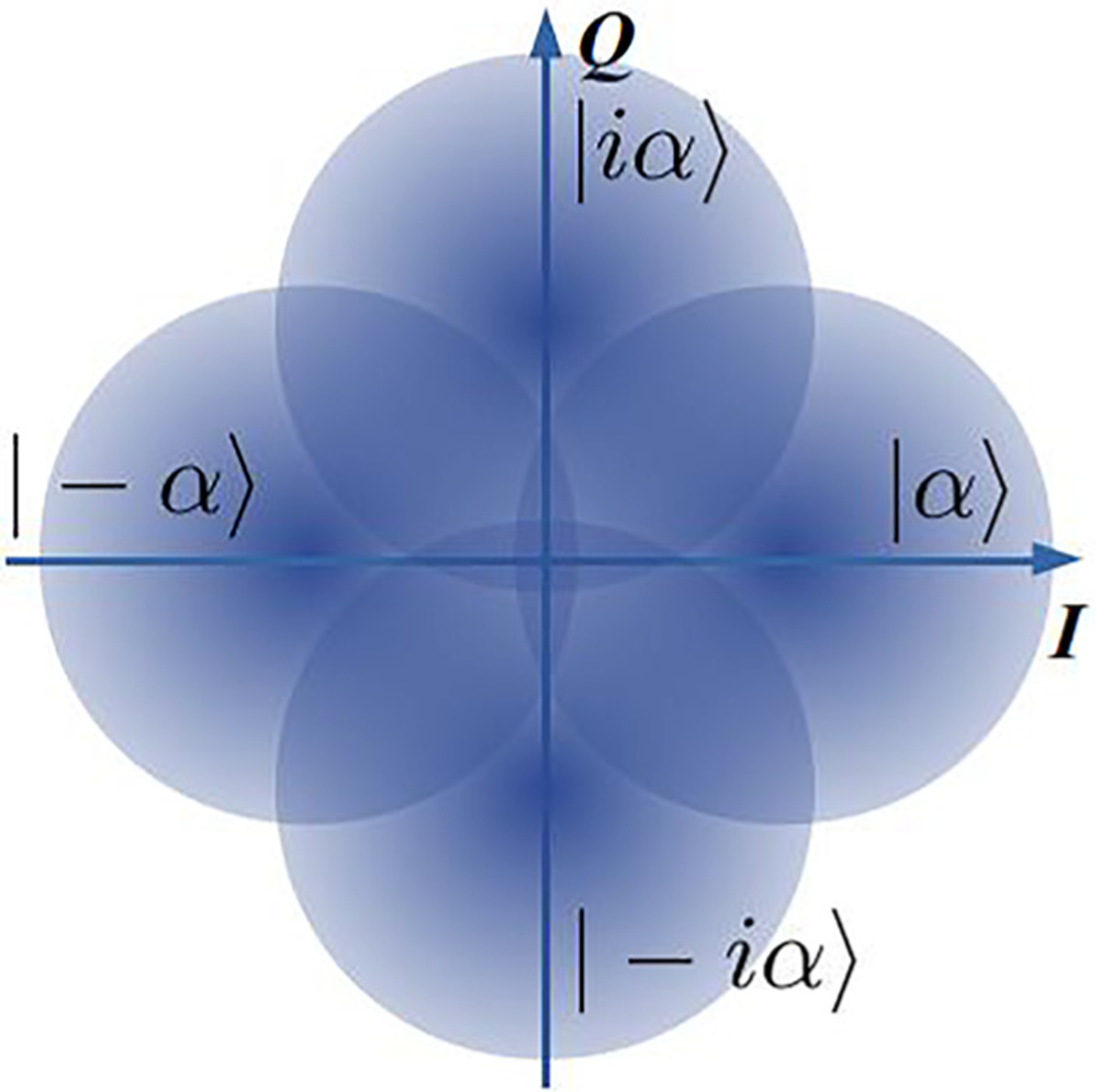
*M*-ary phase shift keying constellation diagram example, *M* = 4.

A natural extension of BPSK is when more than two states are encoded in the phase of a coherent state. From symmetry considerations, the states are separated by equal phases Δ*ϕ* = 2*π*/*M*, where *M* is the number of states in the alphabet. As an example the constellation diagram of the 4-ary PSK is presented in Fig. 4. This modulation method encodes more than one bit per state, which may be beneficial for two reasons. First, when detectors are slow, a single measurement yields several (log_2_*M*) bits, so that the rate of information exchange improves. Second, the number of bits transmitted per optical mode in a unit time is higher; thus, spectral efficiency is higher. SNL and HB can be found analytically in integral form Refs. 23 and 34; see Table II. It is convenient to plot energy and bandwidth requirements of *M*-ary PSK protocols on one graph, where points with different *M* are connected to guide the eye, Fig. 2. Even though *P*_SNL_ > *P*_HB_ for all *M*, error probability bounds for classical and quantum detection grow fast with *M* for a constant energy per bit *n*/log_2_*M*.^34,48^ Unfortunately, the potential advantage of the quantum measurement *P*_SNL_/*P*_HB_ also decreases with *M*. Therefore, quantum receivers are most effective for PSK protocols with relatively low *M* (see SNL PSK and HB PSK in Fig. 2).

### C. *M*-ary orthogonal encodings

**FIG. 5.**
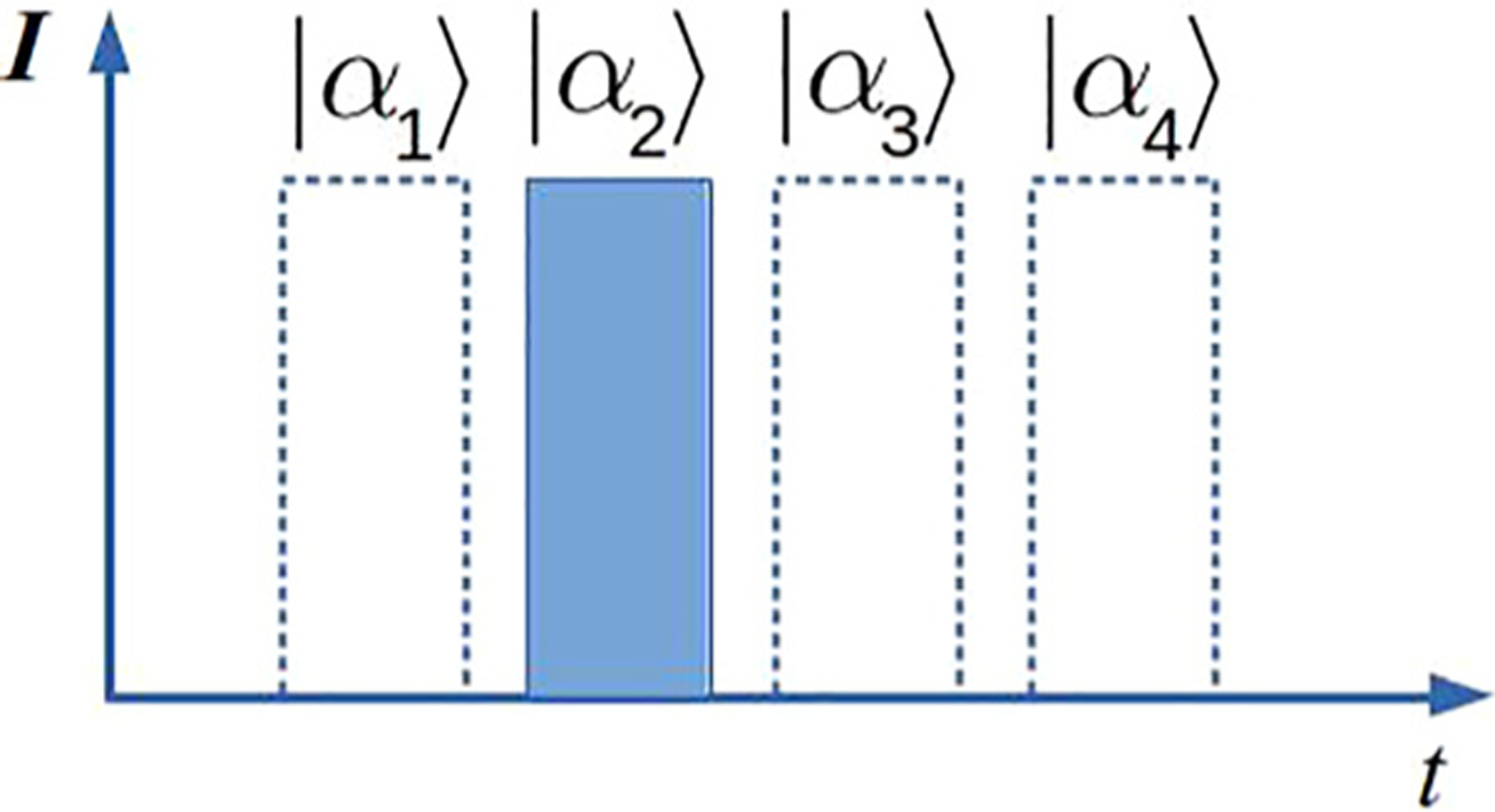
*M*-ary pulse position modulation states, *M* = 4.

The information can be encoded in *M* orthogonal modes, where a single optical pulse occupies one such mode. Modes can be made orthogonal using non-overlapping time bins, non-overlapping frequency bands, polarization, and spatio-angular distributions. Particularly, pulse-position modulation (PPM) is a modulation scheme in which log_2_*M* bits are encoded in one of *M* time bins, Fig. 5. Because different symbols of the alphabet do not overlap in time, this encoding is classically orthogonal. Each time bin can be thought of as an optical mode; therefore, an *M*-ary alphabet occupies *M* modes. Because the duration of a signal is one *T*/*M* time bin, the required bandwidth for this protocol is *M* times broader than that for the flat-top pulse of duration *T*. Instead of using time bins, one can use symbols that are separated in frequency, in which case information will be encoded in spectral modes, and the required bandwidth will still be *M* times broader than that for the flat-top pulse of duration *T*. Linear expansion of bandwidth use is unavoidable for all modulation schemes using orthogonal modes. Other degrees of freedom, such as polarization or spatial modes, can be used when available.

Direct detection is classically the best detection strategy. Specifically, in PPM, modes are separated in time, so the arrival time of the pulse to the detector is sufficient for the physical separation of modes. For other encodings, mode separation may involve spectral filtering, spatial mode sorters, and so on. The classical error limit for ideal signal-shot-noise limited (background-free) detector operation, Table II, is proportional to *e*^−*n*^, i.e., the probability to detect vacuum states in all modes. There is no dependence of *P*_SNL_ on *M* for large *M*. Therefore, for a given power, error per bit reduces with *M* as log_2_(*M*) (DD orthogonal in Fig. 2). This feature is used for photon-starved communications although the energy-bandwidth trade-off becomes inefficient for large *M*.

Even though heterodyne detection is not optimal due to larger shot noise, it is often used in optical communications for orthogonal frequency shift keying. Heterodyne noise increases with the bandwidth. On the other hand, noiseless physical separation of the closely-spaced frequency modes may be practically unfeasible. Interestingly, when heterodyne detection is employed, nearly all gain in bits per unit energy for large *M* is canceled by increasing noise (see SNL OFSK in Fig. 2).

As we discussed above, from the quantum viewpoint, faint coherent states are always nonorthogonal. A Helstrom bound is therefore above zero. Its value can be readily found, Table II (HB orthogonal in Fig. 2), and it can be shown that the *P*_HB_ < *P*_SNL_.^9^ Therefore, orthogonal encoding receivers can also benefit from a quantum measurement.

### D. *M*-ary coherent frequency shift keying

**FIG. 6.**
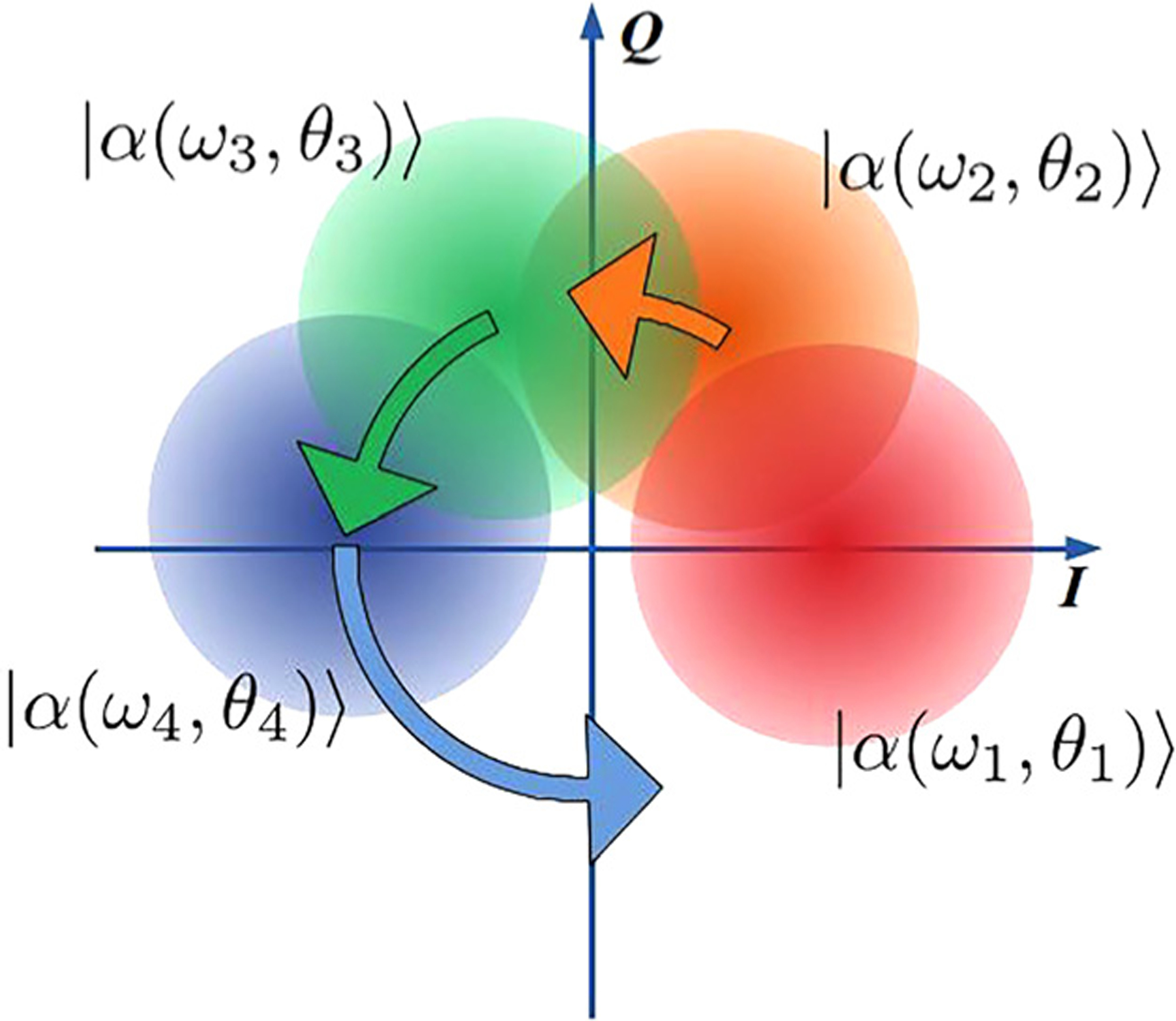
*M*-ary coherent frequency shift keying constellation diagram example, *M* = 4.

The *M*-ary coherent frequency shift keying (CFSK) encodes information in both the frequency and phase of coherent state pulses, |*α*_*m*_〉 = |*α*(*ω*_*m*_, *θ*_*m*_)〉. The adjacent symbols *m* and *m* + 1 are separated by Δ*ω* in frequency space, and their initial phases differ by Δ*θ*, so that |*α*_*m*_〉 = |*α*(*ω*_0_ + (*m* − 1)Δ*ω*, (*m* − 1)Δ*θ*)〉. This alphabet is illustrated in the constellation diagram, Fig. 6. In this diagram, coherent states rotate with time around the origin with rates given by their detuning. The keying can be described by two parameters: Δ*θ* and Δ*ωT*. This parameter space contains the PSK modulation scheme: Δ*ωT* = 0, Δ*θ* = 2*π*/*M* and the orthogonal frequency shift keying (OFSK): Δ*ωT* = 2*π*. The goal here is to reduce the bandwidth of the communication protocol while maintaining low error probabilities. Therefore, one is interested in small frequency separation: Δ*ωT* < 2*π*. In this parameter space, states are nonorthogonal. Therefore, both *P*_HB_ and *P*_SNL_ cannot be expressed analytically. Numerical methods^48,49^ are used instead. Both Δ*θ* and Δ*ωT* can be adjusted to meet certain optimization goals. For instance, when optimizing for energy efficiency, minimal Helstrom bound is achieved with one set of parameters, the lowest shot noise limit requires another parameter set, and the minimal error rate is achieved in a quantum receiver with yet another one. Interestingly, as the numerical analysis of *P*_HB_ shows, this keying balances energy requirements and bandwidth requirements at the same time, for 4 ≤ *M* ≤ 32, see Fig. 2. As a consequence, its rate graph crosses the *R*/*W* = 1 value. Therefore, this keying is neither power limited nor bandwidth limited.

For a properly optimized CFSK $P_{\text{SNL}}^{\text{CFSK}}<P_{\text{SNL}}^{\text{PSK}}$, which is expected, because the bandwidth of CFSK is wider than that of PSK. However, it may be difficult to build an efficient classical CFSK receiver in practice. Interestingly, it turns out that a time-resolving quantum receiver, discussed later, uses the same hardware for many encodings including PSK and CFSK. The only difference is the feedback algorithm encoded in firmware. Therefore, the quantum measurement can be used to provide bandwidth and power efficiency simultaneously in a practical way.

## IV. DISPLACEMENT-BASED QUANTUM STATE DISCRIMINATION

Quantum theory establishes a lower discrimination error bound than that accessible through classical measurement. However, the design of a practical measurement method does not directly follow from theory. In 1973, Kennedy proposed the first near-optimum receiver approaching Helstrom bound for binary coherent states.^17^ In less than a year, Dolinar proposed an improved receiver for binary coherent states.^18^ In both receivers, the input state is displaced from its original state through interference with a local oscillator, which can be practically accomplished with a heavily unbalanced (typically, 99:1) beam splitter. These two seminal papers have triggered theoretical and experimental research of quantum receivers.

Most theoretical and nearly all experimental reports to date take advantage of coherent state displacement in one way or another even though coherent state displacement is not the optimal quantum measurement for some encodings. As it has been shown recently, an optimal projective measurement may require ancillary quantum states or quantum nodes, such as a single atom. We cover this exciting work in Sec. V.

**FIG. 7.**
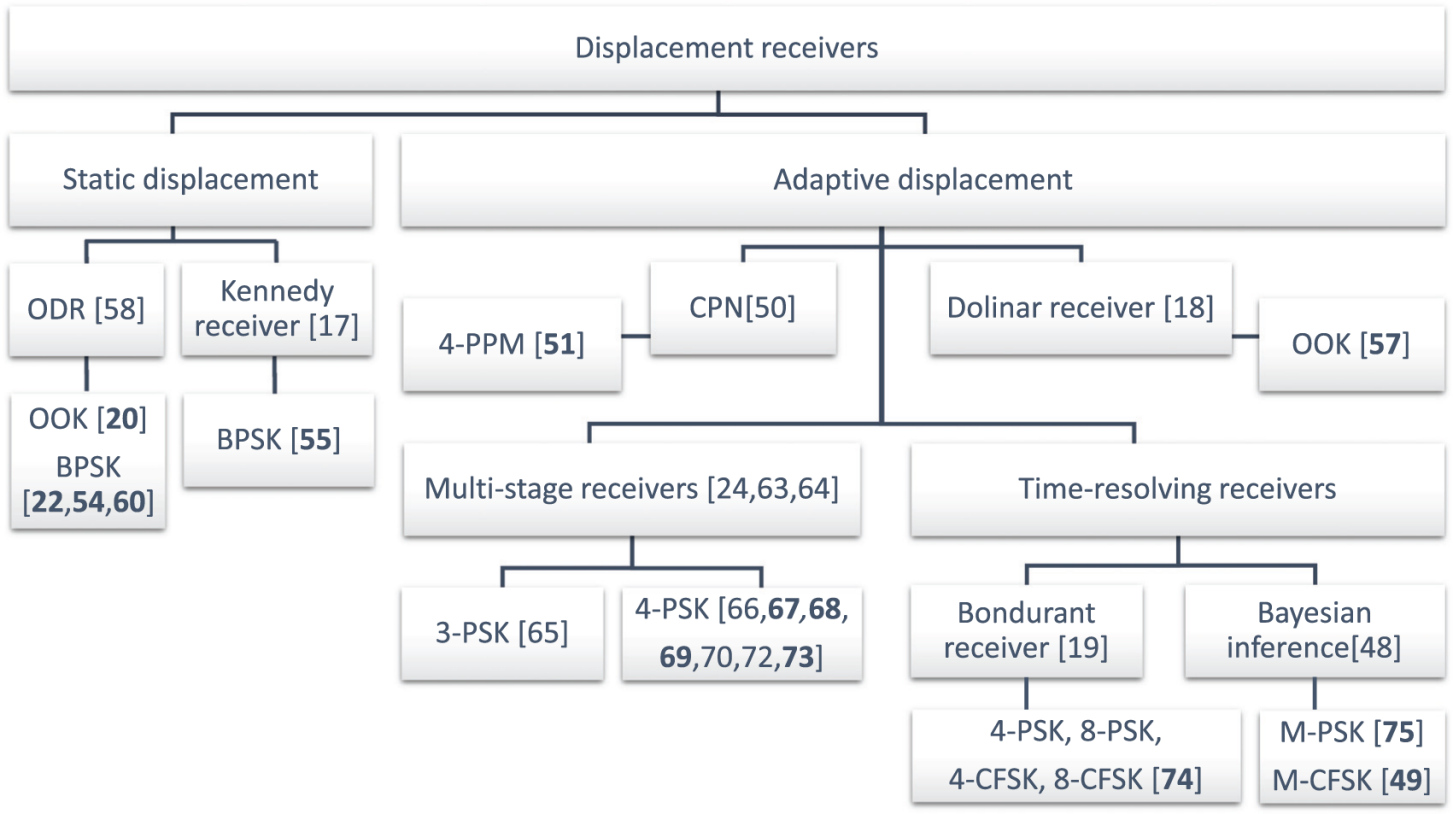
Classification of displacement-based quantum receivers. References to experimental demonstrations are in bold.

In this section, we discuss the experiments with coherent state displacement-based quantum receivers. To aid the reader, we present a simple classification of these receivers in Fig. 7. The classification is based on the principle of operation. Coherent displacement can be either non-adaptive, where the local oscillator does not change throughout measurement (as in a Kennedy receiver) or adaptive, where the coherent state is actively controlled (as in a Dolinar receiver). The lowest level in the figure contains references (in bold) to experimental demonstrations and mentions a modulation protocol(s) used in the experiment.

At the time of writing, we are aware of OOK, BPSK, *M*-ary PSK, *M*-ary PPM, and *M*-ary CFSK experiments. To gauge the performance of quantum receivers, we compiled a table with the experimental results, Table III. The improvement from quantum measurement is typically measured as a ratio of the observed error rate to the classical SNL limit for a noiseless receiver with the same system detection efficiency as the quantum receiver, i.e., adjusted SNL. This measurement quantifies the so-called “quantum advantage” over a classical measurement under similar conditions. However, using this characterization method does not account for any inefficiency of the quantum measurement experiment. Some inefficiencies may be due to imperfect off-the-shelf components that were used, while other inefficiencies may be intrinsic to the chosen quantum measurement method. Thus, one could argue that a more relevant comparison of quantum versus classical receivers is to use the absolute SNL—the limit of the ideal classical receiver with unity efficiency. The error rates below the absolute SNL cannot be achieved by a classical receiver in principle. Although all quantum receivers surpass the adjusted SNL, not all of them achieve SER below the absolute SNL. We also compare input state energy required to achieve SER of 10% for demonstrated quantum receivers versus the SNL-limited receivers where applicable. This comparison shows the possible reduction of energy requirements by switching to quantum receivers.

### A. Kennedy receiver

**FIG. 8.**
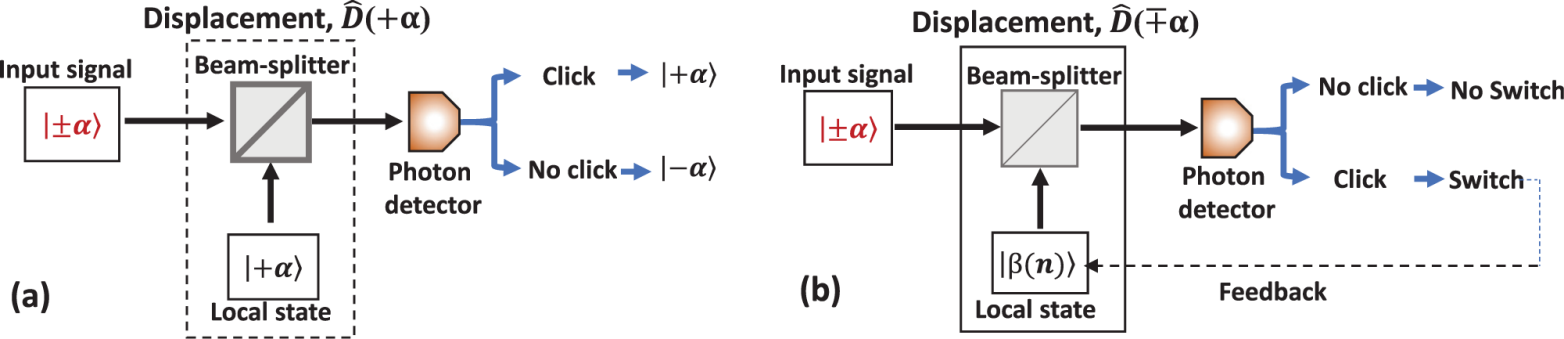
Schematic diagram of first quantum receivers for binary state discrimination. The displacement operation, $\hat{D}$, uses a local oscillator state and a beam-splitter. (a) Kennedy-like receiver (non-adaptive) and (b) Dolinar-like receiver (with feedback).

Helstrom determined the fundamental SER bound for the optimum receiver in 1968,^10^ where the projection measurement on a quantum superposition state, often called “Shrödinger cat state” was proposed to reach the quantum limit for the binary coherent state encoding. The experimental implementation of the proposed optimal measurement is very difficult because it relies on a superposition basis and entanglement measurements.^52^ This method requires a very high-fidelity entanglement and a near-unit detection efficiency.^53^ In 1973, Kennedy proposed the first receiver using a simple displacement operation on the input coherent state followed by photon detection.^17^ While the overall performance of the receiver falls short of the HB, the receiver achieves exponentially optimum performance and outperforms the shot noise limit.^17^ The receiver scheme proposed for BPSK states | + *α*〉 and | − *α*〉 is shown in Fig. 8(a). The input signal is displaced using a local coherent state and measured using a photon detector. The displacement occurs by interfering with the input signal with the local state on a beam-splitter. As shown in Fig. 8(a), the local state is set to | + *α*〉. The destructive interference occurs for the input signal | − *α*〉 which is displaced to vacuum |0〉, so no photon can be detected. The constructive interference occurs for | + *α*〉, such that the output is displaced to | + 2*α*〉. A brighter output makes the probability to detect a photon higher. Therefore, in the ideal noiseless case and with the perfect displacement no photons will be detected when the input state was | − *α*〉, but there is a probability (proportional to exp (− 4|*α*|^2^)) that no photons will be detected if the input state was | + *α*〉. This non-zero probability causes a discrimination error. In spite of the apparent simplicity of the method, experimental implementations^54,55^ fell short from outperforming the absolute SNL due to low system efficiency, non-ideal displacement, and dark noise at the detector. Modified Kennedy receivers use an optimized displacement and a more sophisticated discrimination algorithm. Those receivers unconditionally surpass the SNL in experiments,^20,22^ discussed below.

### B. Dolinar receiver

Following the proposal of the first quantum receiver using non-Gaussian measurements to beat the shot-noise limit, Dolinar proposed a receiver^18^ that can reach the Helstrom bound for discrimination of binary coherent states. This receiver theoretically approaches the quantum limit in binary state discrimination by using the real-time quantum feedback with the so-called optimal displacement and photon counting measurements, i.e., without the need for a “cat-state” measurement.^10^ In contrast to the Kennedy receiver, the displacement amplitude *β* is changing constantly. The phase is adjusted every time a photon is detected, i.e., it is determined from the number of photons *n*_*t*_ detected in the time interval [0, *t*).^18,56^ For an on–off keying, the optimal displacement amplitude is given by^57^

(12) $$\beta \left(n_{t}\right)=\frac{\alpha }{2}\left[\frac{e^{i\pi \left(n_{t}+1\right)}}{\sqrt{1-e^{-|\alpha {|}^{2}t/T}}}-1\right].$$

The discrimination decision is based on the total number of photons *n*_*T*_ counted during the entire measurement [0, *T*], so that |*α*〉 (| − *α*〉) is chosen when *n*_*T*_ is even (odd) as shown in Fig. 8(b). Formally, Eq. (12) diverges at the beginning of the pulse *t* = 0, which cannot be practically implemented because of the finite energy of the LO and the saturation of a single-photon detector.

Yet, this issue can be practically alleviated in a laboratory environment. A binary Dolinar-like receiver with finite displacement amplitudes was successfully implemented experimentally in Ref. 57. In their work, authors demonstrated that for input signal with the low average number of photons (*n* <1) the OOK receiver not only surpasses the adjusted SNL, but also approaches the adjusted HB; for comparison, both SNL and HB were adjusted to the system efficiency.

Dolinar’s idea of adaptive feedback enabled multiple new quantum receiver configurations. Particularly, sub-SNL receivers for *M*-ary encodings were invented and experimentally demonstrated.

### C. Novel quantum receivers and experiments

#### 1. The optimized displacement receiver

**FIG. 9.**
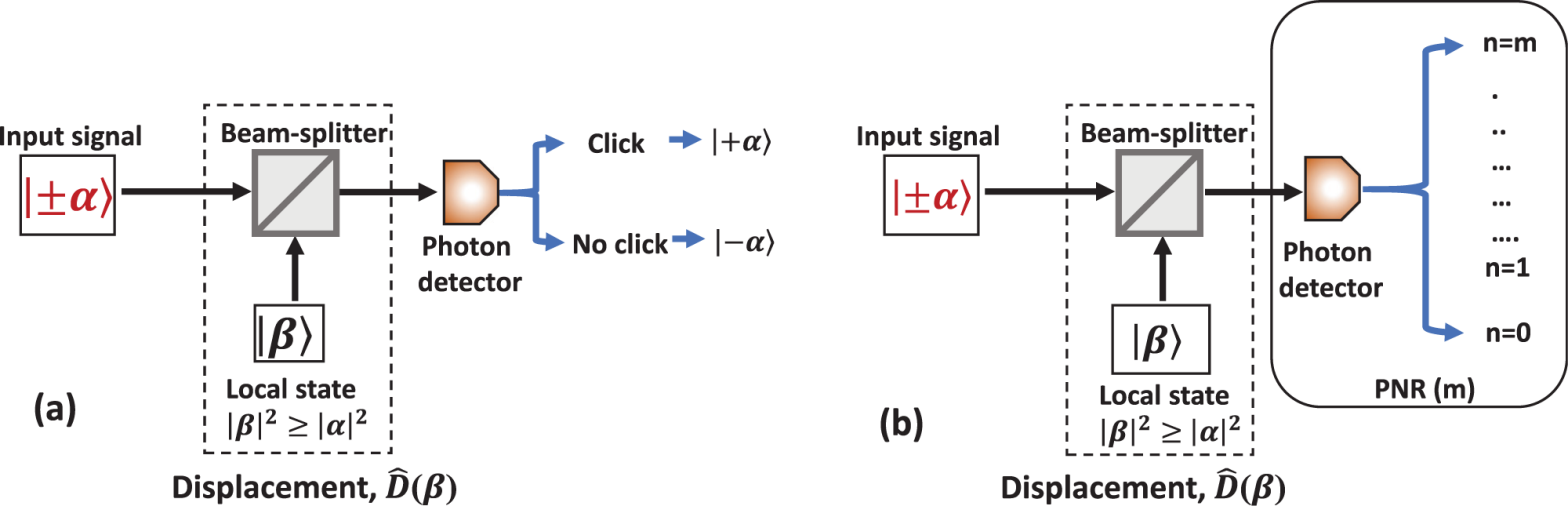
Schematic diagram of modified Kennedy receivers. (a) Optimal displacement receiver (ODR) and (b) ODR with photon number resolution (PNR).

A few attempts were made to modify Kennedy receivers to achieve a lower SER. One such enhancement is the optimized displacement receiver (ODR). Kennedy receiver displaces the input state by interfering it with the equal amplitude of the local state. In their theoretical paper, Takeoka and Sasaki proposed to adjust the displacement of the input signal using local state.^58^ Their ODR uses the local state with an amplitude *β* greater than the input signal amplitude *α*, Fig. 9(a). It is evident that due to unequal amplitude in the local state the input signal will not be displaced to vacuum. There are no other changes to the Kennedy design, cf. Fig. 8(a). Since larger displacement results in a higher probability of photon detection when input signal state is displaced to |*α* + *β*〉, the probability of detecting no photons $e^{-|\alpha +\beta {|}^{2}}$ is reduced from that of the Kennedy receiver. However, because | − *α*〉 is no longer displaced to vacuum, there is a possibility to collect photons, which leads to errors. The trade-off between these “false” detections due to the non-ideal vacuum |*β* − *α*〉 and the reduced probability to get no clicks for the |*β* + *α*〉 state results in an optimization problem. The optimal displacement amplitude *β* minimizes the combined error probability. The experimental implementations of ODR has shown discrimination error rates below the SNL adjusted for the experimental conditions^54,59^ and unconditionally,^20,22^ i.e., in comparison to the absolute SNL. The most significant improvement in discrimination accuracy is shown for faint coherent states with |*α|*^2^ ≈ 1. The amplitude of the optimized displacement approaches the amplitude of the input state |*β*| → |*α*| as |*α*| → ∞. A similar optimization of displacement can reduce the discrimination error rate of adaptive feedback receivers for binary and *M*-ary alphabets as well.

The discrimination error rate of the ODR receivers can be further reduced with photon-number resolving (PNR) measurements [Fig. 9(b)] and can extend the below-SNL performance of the receiver to higher input energies |*α*|^2^.^22,60^ A discrimination threshold is the particular number of detected photons during *T*. If the total number of detections is below that threshold, | − *α*〉 is received; otherwise, |*α*〉 is received. Note that with a notable exception of Refs. 20 and 22 where transition edge sensor (TES) detectors were employed, other receivers use a quasi-PNR detector. A conventional single-photon avalanche photodiode’s (SPAD) clicks are counted. The total count of clicks corresponds to the number of photons to within the detector’s deadtime, afterpulsing, and dark count probability.^27,61,62^

#### 2. Conditional pulse nulling receiver

**FIG. 10.**
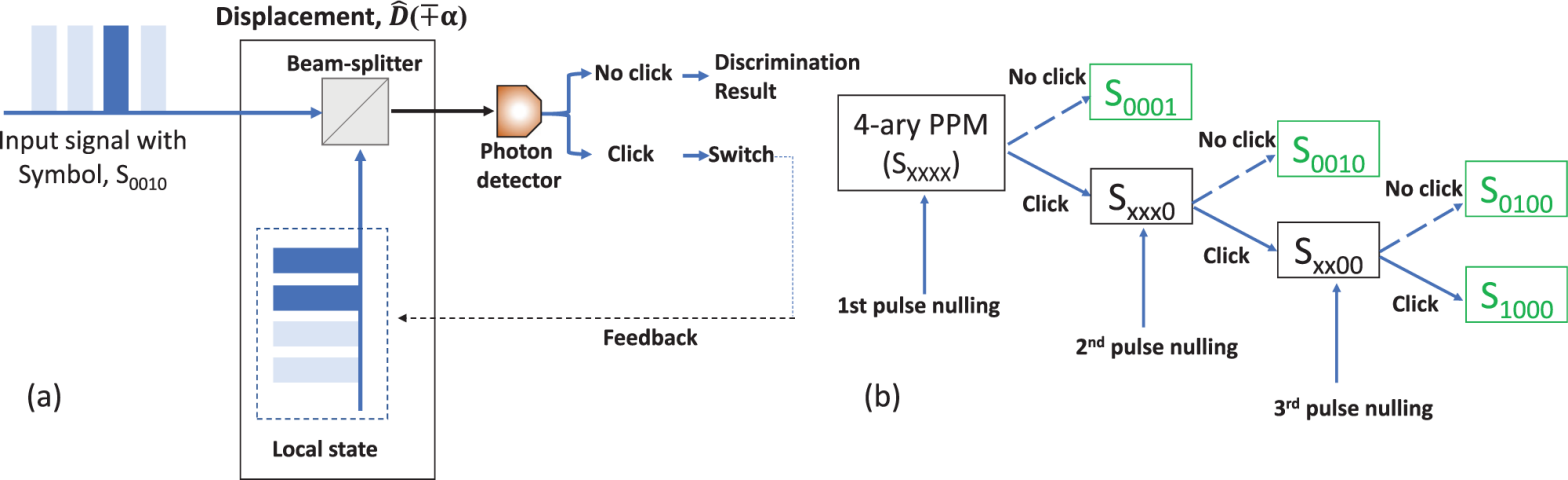
(a) Experimental scheme of CPN receiver for 4-ary PPM. (b) Decision strategy for 4-ary PPM. Broken arrows represent no photon detection and solid arrows represent photon detection after nulling. Green boxes are the received states after discrimination.

Conditional pulse nulling (CPN) receivers are explicitly designed for pulse position modulation (PPM) which is widely used in photon-starved free space communications due to its high energy efficiency. Dolinar proposed the CPN receiver in 1982.^50^ He theoretically showed that CPN performs near the optimum.^50^ Almost three decades later, the CPN receiver has been experimentally demonstrated for a 4-ary PPM with the discrimination error below the adjusted SNL.^51^ The experimental scheme of the CPN receiver is shown in Fig. 10(a). The input signal is displaced to vacuum using the local state pulse. The decision strategy for 4-ary PPM is shown in Fig. 10(b). The receiver starts by nulling the pulse in position 1 (Fig. 5). Photon detection (failure) leads to the nulling of pulses in the subsequent steps. If no photons were detected in position 1 (success), then the received state is |*α*_1_〉. The same strategy is repeated for subsequent positions. The green boxes represent the received state after a discrimination. Even in ideal experimental conditions, errors arise from the Poisson nature of the coherent states, cf. Kennedy receiver: the displacement of the input signal with a wrong local state does not necessarily lead to photon detection.

#### 3. Multi-stage receivers

The optimal receiver for binary coherent states proposed by Dolinar^18^ requires feedback to adjust the LO as more information about the input state becomes available. A possible modification of the Dolinar receiver that makes it more experimentally feasible breaks the input into segments or stages either spatially [(11(a)] or temporally [(11(b)]. Then, the measurement result from each segment can be used to choose the best displacement state for the next segment. The number of stages is predefined. Switching rules can be represented as a decision-making tree that is typically precomputed. It can be shown^63,64^ that with the proper choice of the displacement intensity at each stage *n* (|*β*_*n*_*|*^2^ > |*α*_*i*_*|*^2^, cf. Dolinar receiver) and in the limit of infinite number of stages such a multi-stage receiver can optimally discriminate binary states. Thus, choosing the same intensity of the LO for all stages does not enable the HB-limited discrimination even when the intensity is optimized.

**FIG. 11.**
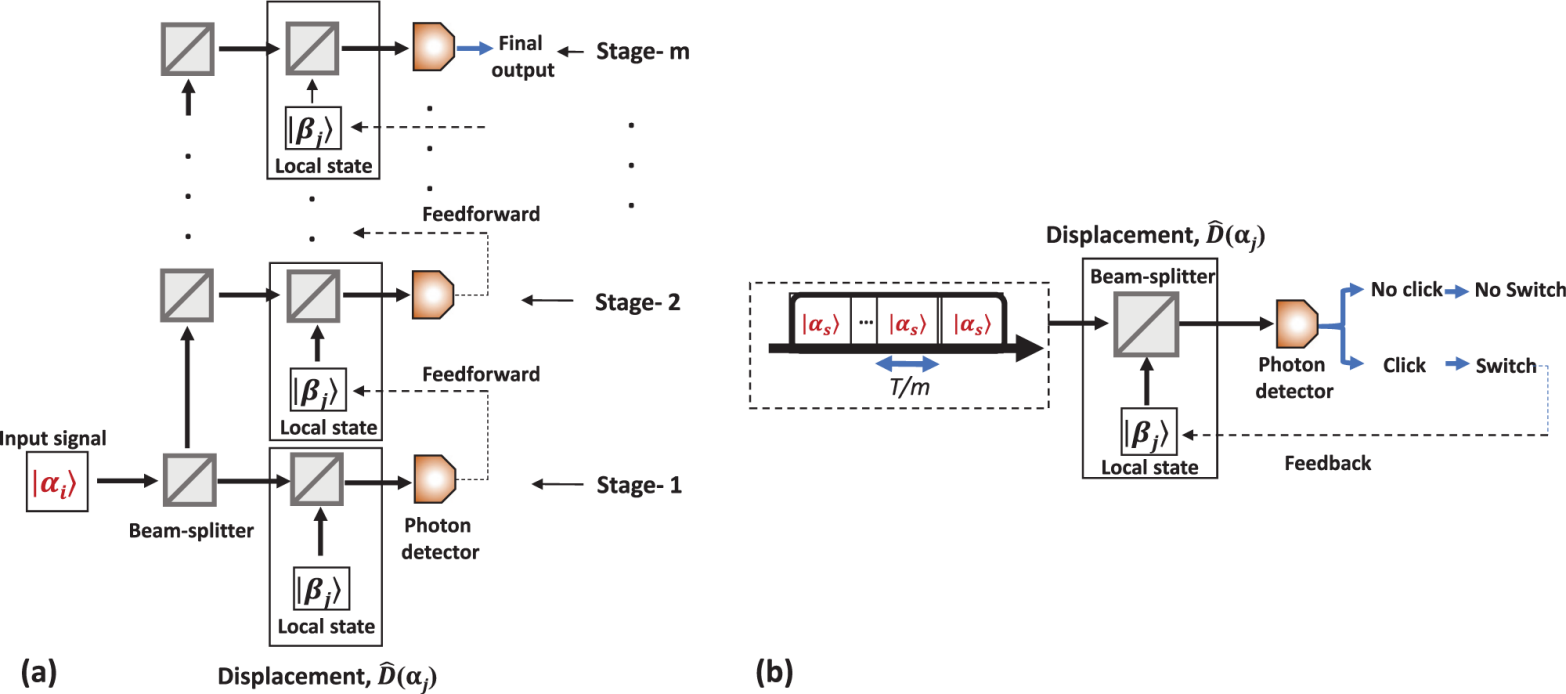
Schematic diagram of adaptive displacement receivers: (a) spatial adaptive displacement receiver and (b) temporal adaptive displacement receiver.

For example, the BPSK input state, |*α*_*i*_〉, is split into multiple copies with equal intensity, Fig. 11(a). Thus, the energy of the input to each stage is reduced by the factor of *m*. Each attenuated copy of the state is sent to a displacement setup. An optical delay is inserted in each stage so that the measurement on an *n* + 1th stage does not start before the measurement on the *n*th stage is completed. For the first stage, an arbitrary state of the LO is chosen. If the LO matches the input, the input state is displaced to vacuum, no photons will be detected; otherwise, a photon can be detected. To achieve close to optimal performance, the value of |*β*_*n*_|^2^ should be corrected at each stage, but the phase of |*β*_*j*_〉 only changes with photon detection. The potential drawback of this scheme is that the number of optical elements and single-photon detectors grows with the number of stages. An excessive loss of the optical signal occurs due to imperfect optical components. In addition, the alignment of the multistage setup may be complicated.

A signal pulse can be divided into equal temporal intervals rather than spatially. In this case, just one LO with the feedback and one detector is needed. As before, the feedback is used to update the LO after each measurement segment with an equal duration *T*/*m*. Figure 11(b) shows the experimental scheme of the multi-stage receiver with temporal stages. The strategy tests the hypothesis that the most probable input signal is *α*_*i*_ during each measurement segment. At the end of the signal pulse *T*, final Bayesian probabilities are computed, and then the hypothesis with the highest probability is used to make the discrimination decision. The main drawback of temporal segmenting is the need for faster detectors and electronic components. A deadtime of single-photon detectors is yet another obstacle.

The idea of adjusting the feedback after each photon detection can be generalized for *M*-ary communication protocols although the optimal feedback algorithm is not known. An *M*-ary discrimination strategy that uses *m* measurement stages where LO can be adjusted after each stage was proposed in Ref. 24. In this paper, the signal is split on a beam splitting tree, where each measurement stage has its own LO and detector. Formally, the Bayesian probabilities for the possible input state are calculated based on the outcome of the measurement (click or no click) in each stage. Bayesian probabilities are used to set the LO of the next stage to verify the most probable hypothesis. Finally, after all *m* measurement results are known, the Bayesian probabilities are updated one last time and the most likely hypothesis is used as the discrimination outcome. This proposal does not consider optimizing the LO intensity separately for each stage. The spatial multi-stage approach was further investigated theoretically for *M* = 3, 4 PSK in Ref. 65. In Ref. 66, authors investigate the theoretical performance of the 4-PSK receiver by adding PNR capabilities.

Temporal adaptive receivers can also be generalized to longer alphabets, Fig. 11(b). The temporal adaptive receiver design was used in the experimental demonstration of the 4-PSK quantum receiver that unconditionally surpassed the SNL limit.^67^ A similar design was used for the first demonstration of the 4-PSK receiver at a telecom wavelength.^68^ A more sophisticated version of this receiver counts the number of photons in each measurement. This approach enables more precise Bayesian calculations and especially helps with sub-SNL measurements of mesoscopic input states. The information about the number of detected photons is particularly helpful against the experimental imperfections such as darkcounts, non-ideal visibility, etc. Thus, lower discrimination error probability can be achieved. In Ref. 69, a SPAD-based quasi-PNR detection was used. The authors extended the sub-SNL performance of their receiver to the inputs with more than 20 photons per pulse on average.^69^ They achieved the record SER (below 10^−6^). The similar quasi-PNR enhancement with a SPAD detector was used to optimize other multi-stage receivers.^66,70–72^ Adjusting intensity of the LO is yet another path to sensitivity improvement. In Ref. 73, the theoretical model of displacement for *M*-ary receivers is optimized by optimizing |*β*|^2^ at each step and the unconditional error rate below signal-to-noise ratio (SNR) is experimentally demonstrated.

#### 4. Time-resolving receivers

Another class of receivers consists of one displacement module and one single-photon detector and uses single-photon detection times for discrimination. Unlike multi-stage receivers, it provides instantaneous feedback to switch the LO state right after each photon detection. By design, the receiver gets to test the unrestricted number of hypotheses and allocates the optimal time to verify each hypothesis. Owing to the nature of coherent states, with a sufficiently fast detector, the probability to detect more than one photon in the field is negligible. Therefore, PNR detection is not required.

**FIG. 12.**
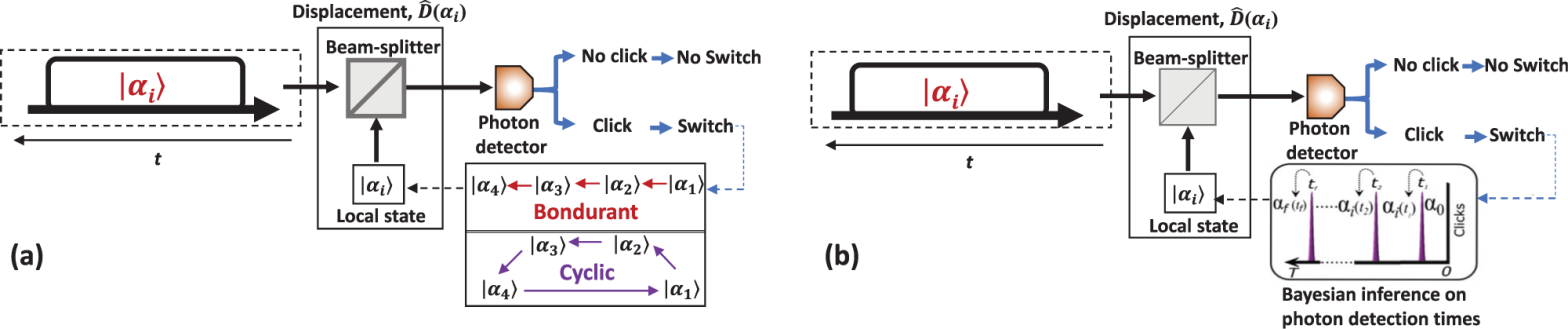
Time-resolving adaptive displacement receivers: (a) Bondurant/cyclic receiver and (b) time-resolving receiver with Bayesian inference.

The first receiver of this class was introduced by Bondurant.^19^ Type-I Bondurant receiver probes hypothesis in a simple sequential order and uses the hypothesis at time *T* as the discrimination decision, Fig. 12(a), while Type-II receiver uses the sequential order, but compares photon interarrival times to make the final discrimination decision. Bondurant receivers have a near-optimal performance for 4-PSK state discrimination, where a Type-II receiver outperforms the Type-I receiver at low input energies. The probing is executed by switching the local state from one hypothesis to next, *α*_1_ → *α*_2_… → *α*_*m*_, until all hypotheses are tested or no more clicks are detected. In a practical setting, a detection event can be induced by a dark count or non-ideal displacement. After any photon detection, the Bondurant receiver discards the hypothesis and will never test it again, leading to extra errors. A cyclic strategy can correct some of these errors. A cyclic receiver is similar to the Bondurant Type I receiver, except after testing the last state of the alphabet *α*_*M*_ it switches back to the first state *α*_1_ and continues the measurement until the end of the pulse *T.*^26^ The cyclic receiver was demonstrated experimentally.^74^ The measured SER is unconditionally better than the SNL for 4-PSK, 8-PSK, 4-CFSK, and 8-CFSK encodings.

A much better result can be obtained if the time-resolving quantum receiver uses both instantaneous feedback and Bayesian inference.^48^ A Bayesian classifier uses the knowledge about prior local state and a photon arrival time to predict the most probable input state after each photon detection. This strategy converges to the right hypothesis with a minimal number of photon detections and it can be applied to any encoding.^75^ The strategy works best if the encoding is developed to take advantage of the instantaneous feedback.^48,49^ This holistic approach when both the receiver and the encoding are developed side-by-side has resulted in the record low error rates in discrimination of large alphabets with faint signals (|*α*|^2^ ≲ 1 photon per bit). This receiver is shown to perform unconditionally below the SNL for *M* ≤ 16 alphabets, the largest number of states in an alphabet reported to date.^49^

### D. Summary of displacement receivers

In summary, a direct comparison of different displacement receivers is not always possible. For binary protocols, the optimal measurement is theoretically possible; measurement schemes that are asymptotically optimal have a clear advantage. For longer alphabet lengths, displacement measurements are not optimal. Theoretically, time-resolving protocols and the protocols that adjust LO intensity throughout the measurement are the most advantageous.

In experiment, practical considerations may play the decisive role. In general, based on experimental evidence, Table III, the protocols that take advantage of photon number resolution perform particularly well for brighter input states. Time-resolving protocols perform better with dimmer input states (with ≈ 1 photon/bit and lower). This is because detectors have deadtime and the feedback components have latency; therefore, fewer feedback cycles may be practically advantageous. Other considerations include the following:

• Transmission loss and detection efficiency. Both properties reduce system efficiency and reduce the unconditional advantage over the absolute SNL.
• Alignment of the displacement reduces both conditional and unconditional advantage of the quantum measurement, but can be partially mitigated by including the inefficiency into the feedback model.
• Background and dark counts similarly reduce both conditional and unconditional advantage of the quantum measurement, and can be partially mitigated by adjusting the feedback model.

We see that spatial multiplexing can remedy time delays, but it may introduce higher losses and alignment issues. The choice of the most optimal modulation protocol and the alphabet length may also depend on experimental and/or practical conditions. In making the choice, considering both conditional and unconditional performance of a receiver (Table III) is important because the conditional performance shows the degree of the advantage made specifically by a non-classical measurement whereas the unconditional performance reveals the system efficiency penalty.

## V. NEW TRENDS

In Sec. IV, we discussed theoretical and experimental achievements in coherent state discrimination with displacement-based quantum receivers. The field of quantum measurement is very active, and many new ideas for using quantum measurement in optical networks have emerged. Here we briefly discuss new research directions that in our view have a significant practical potential.

### A. Noisy communication channels

**FIG. 13.**
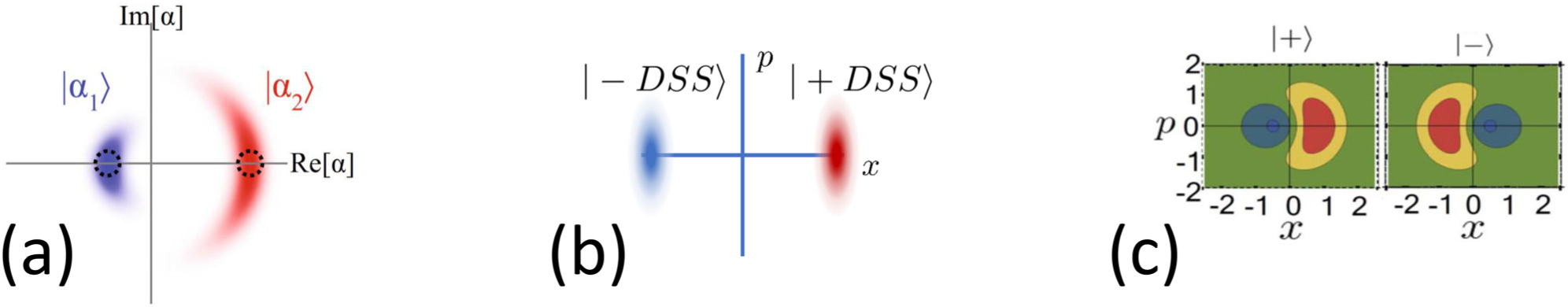
(a) Binary signals optimized for communication in the channel with phase diffusion. Reprinted with permission from DiMario *et al.*, npj Quantum Inf. **5**, 65 (2019). Copyright 2019 Authors, licensed under a Creative Commons Attribution 4.0 International License;^76^ (b) displaced squeezed states (DSS) with opposite phases, as described in Ref. 79; (c) Wigner functions of single-rail qubit states, reproduced with permission from Izumi *et al.*, J. Phys. B **51**, 085502 (2018).^80^ Copyright 2018 IOP Publishing. All rights reserved.

Realistic communication channels may distort and contaminate communication signals. Given that the theory of quantum receivers assumes noiseless channels, it is important to understand if quantum measurement advantage extends to channels with noise. An important realistic channel model is the non-Gaussian channel with bosonic phase noise. In Ref. 76, authors investigate a communication strategy over channels with phase noise and demonstrate that quantum measurement may be advantageous. In particular, authors optimize the displacement of the BPSK signals by varying LO amplitude, cf. Ref. 58, paired with a Kennedy-like receiver that takes advantage of PNR, cf. Ref. 60 [Fig. 13(a)]. They demonstrated SER below the homodyne limit adjusted for the system efficiency of 72% in the presence of phase noise. A similar strategy for a channel with thermal noise is considered in Ref. 77. The authors theoretically demonstrate that a PNR-enabled Kennedy-like receiver with the optimized displacement (see Refs. 58 and 60) can surpass the SNL when the average number of thermal photons is smaller than 0.2. Practical implementations of many quantum receivers require interferometric stability of the communication channel or a pilot signal providing the reference phase. In long-distance communication, it may be challenging to interferometrically stabilize the communication channel. In Ref. 78, authors experimentally demonstrate a phase-tracking protocol for quantum receivers to correct for time-varying phase noise and keep SER below the SNL.

### B. Discrimination of optical states other than coherent states

So far we considered coherent states as communication carriers. This is because coherent states of light are widely used for communication. Other types of states can be discriminated using quantum methods as well. The optimal discrimination of optical states with non-Poissonian photon number statistics^81–84^ has recently attracted a lot of interest. In these new experiments, ancillary coherent states are used for displacement in a receiver. Clearly, the perfect displacement of a non-Poissonian state to a vacuum state with a coherent state is impossible. Still, the probability to detect at least one photon can be significantly increased for one type of input and significantly reduced for the other.

In Ref. 79, authors investigate a binary communication channel that uses squeezed vacuum states as information carriers. The information is encoded by displacing the squeezed vacuum state by $\hat{D}(\pm \alpha )$,^16^ resulting in two displaced squeezed states (DSS) |±DSS〉 with the opposite phases [cf. BPSK, see Fig. 13(b)]. Squeezing of one of the quadratures of the carrier gives a smaller overlap between the DSS states in comparison to coherent states with the same average number of photons. Thus, the discrimination error probability for the squeezed states, in theory, may fall below the Helstrom bound for BPSK with coherent states in the absence of loss. When the channel has some phase noise, but no significant loss, even a homodyne-based “classical” receiver can approach the quantum optimum.

In Ref. 85, the fundamental quantum limit for discrimination error probability between a coherent and a thermal optical state is computed. Additionally, error probability bounds for direct detection, coherent homodyne detection, and the Kennedy-like receiver are given. The generalization of the Kennedy receiver for discrimination of coherent and thermal states with a low average photon number is shown to closely approach the quantum limit.

**FIG. 14.**
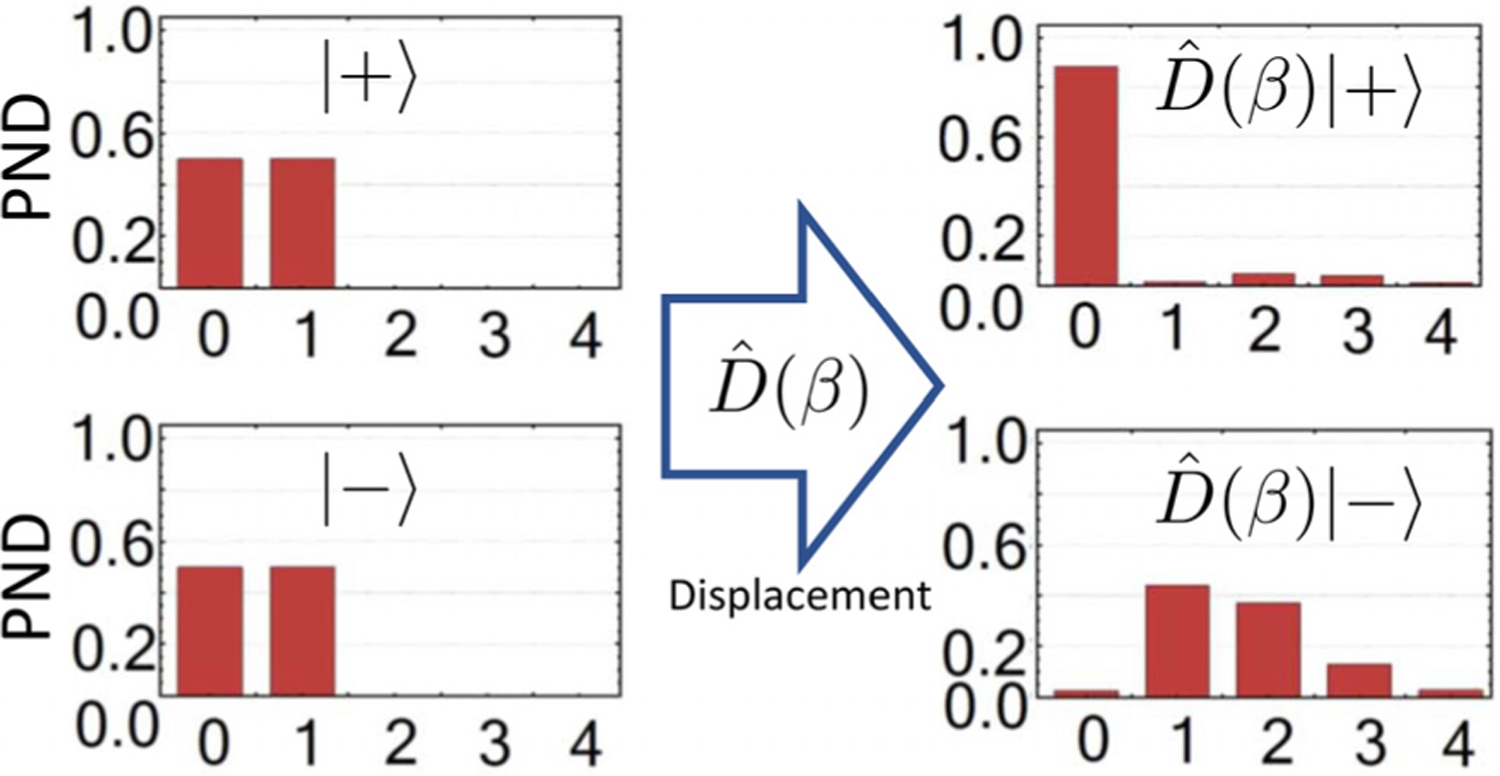
Photon number distribution of single-rail qubits before and after displacement, reproduced with permission from Izumi *et al*., J. Phys. B **51**, 085502 (2018).^80^ Copyright 2018 IOP Publishing. All rights reserved.

The displacement-based discrimination strategies used by quantum receivers were recently adopted for the discrimination of single-rail qubits, a superposition of the vacuum state with a single photon. In Refs. 80 and 86, authors theoretically and experimentally investigate a receiver for orthogonal single rail qubits: $|\pm \rangle =(|0\rangle \pm |1\rangle )/\sqrt{2}$[see Fig. 13(c)]. Authors have shown that their setup can discriminate the superposition states using weak coherent states for displacement. Both input states have a certain vacuum and single-photon components. After coherent state displacement, the resulting states have distinct photon-number statistics, Fig. 14. This difference in mean photon numbers can be assessed with a single-photon detector. A feedback discrimination strategy generalized for single-rail qubits yields an SER below that of the perfect homodyne detection. These results can facilitate the implementation of quantum information processing protocols using single-rail qubits.

### C. Quantum unambiguous state discrimination

Displacement-based quantum receivers can be employed for so-called unambiguous state discrimination (USD).^71,87,88^ Unlike a typical receiver whose goal is to provide the best guess for all input states, unambiguous state discrimination receivers aim to error-free discrimination of states or reject the measurement as inconclusive if that cannot be done. In Ref. 71, sub-SNL USD is experimentally demonstrated for BPSK. At a later time, sub-SNL USD was extended to 4-PSK in Refs. 87 and 88.

### D. Optimal quantum measurements

We saw that displacement receivers are optimal for some encodings. For other encodings, displacement receivers cannot reach the HB. There is an alternative to displacement measurements, however. For instance, an optimal projective measurement with the help of quantum states, such as cat states,^89^ has been proposed and experimentally implemented. To our knowledge, this work is the only experimental effort to date that enables a quantum receiver that is not based on coherent state displacement. Yet another idea is to take advantage of an ancillary quantum system, such as a single atom.^90–92^ In these proposals, the input light field is mapped on a discrete set of atomic states, followed by a projection measurement. Near-optimal discrimination of BPSK, *M*-PSK, and *M*-ASK (amplitude shift keying) encodings has been discussed. An efficient light field interaction with an ancilla atom is required, which may be challenging to experimentally implement with today’s technology. Another theoretical proposal shows how to design the optimal receiver for an arbitrary alphabet length and an arbitrary modulation scheme with the help of a universal quantum computer. The input signal is split to *m* copies each of which is transferred to the quantum computer. The quantum computer performs *m* unitary operations on the ancilla quantum register. The final state of the ancilla register is measured to arrive to the discrimination result.^93^ This idea uses two properties of coherent states: first, splitting a coherent state produces coherent states with the same properties, except for amplitudes; second, a coherent state with a sufficiently small amplitude is well approximated by a single-rail qubit (cf. Refs. 80 and 86). The problem of discriminating coherent states is reduced to discriminating multicopy single-rail qubit states by a sequential coherent-processing receiver.^94^

### E. Artificial intelligence in communication

One of the interesting future directions for quantum receivers is the possible use of the artificial intelligence for real-time feedback and discrimination. Recently, artificial neural networks were successfully applied to reduce the error probability of the classical communications system, achieving the classical optimal limit.^95^ Replacing or pairing Bayesian inference with artificial neural networks could optimize feedback strategy and reduce error rates of quantum receivers in practical settings.

## VI. THE QUANTUM MEASUREMENT ENHANCED CLASSICAL INTERNET OF THE FUTURE? (IN LIEU OF CONCLUSION)

As it is evident by now, below-the-shot-noise limit discrimination error rates for coherent states have been achieved in many laboratories and for different encoding methods. Properties of displacement-based quantum receivers using non-Gaussian measurement were extensively studied. The field, however, is still in its early stage. Indeed, just one experimental report achieved SERs unconditionally below the classical limit at a telecom wavelength,^68^ while other proof-of-principle experiments either use visible light or cannot unconditionally surpass the SNL.^55^ Conventional communication systems, on the other hand, are very successful, mature, and competitive. Let us discuss the possible future of quantum technologies for classical communication.

**FIG. 15.**
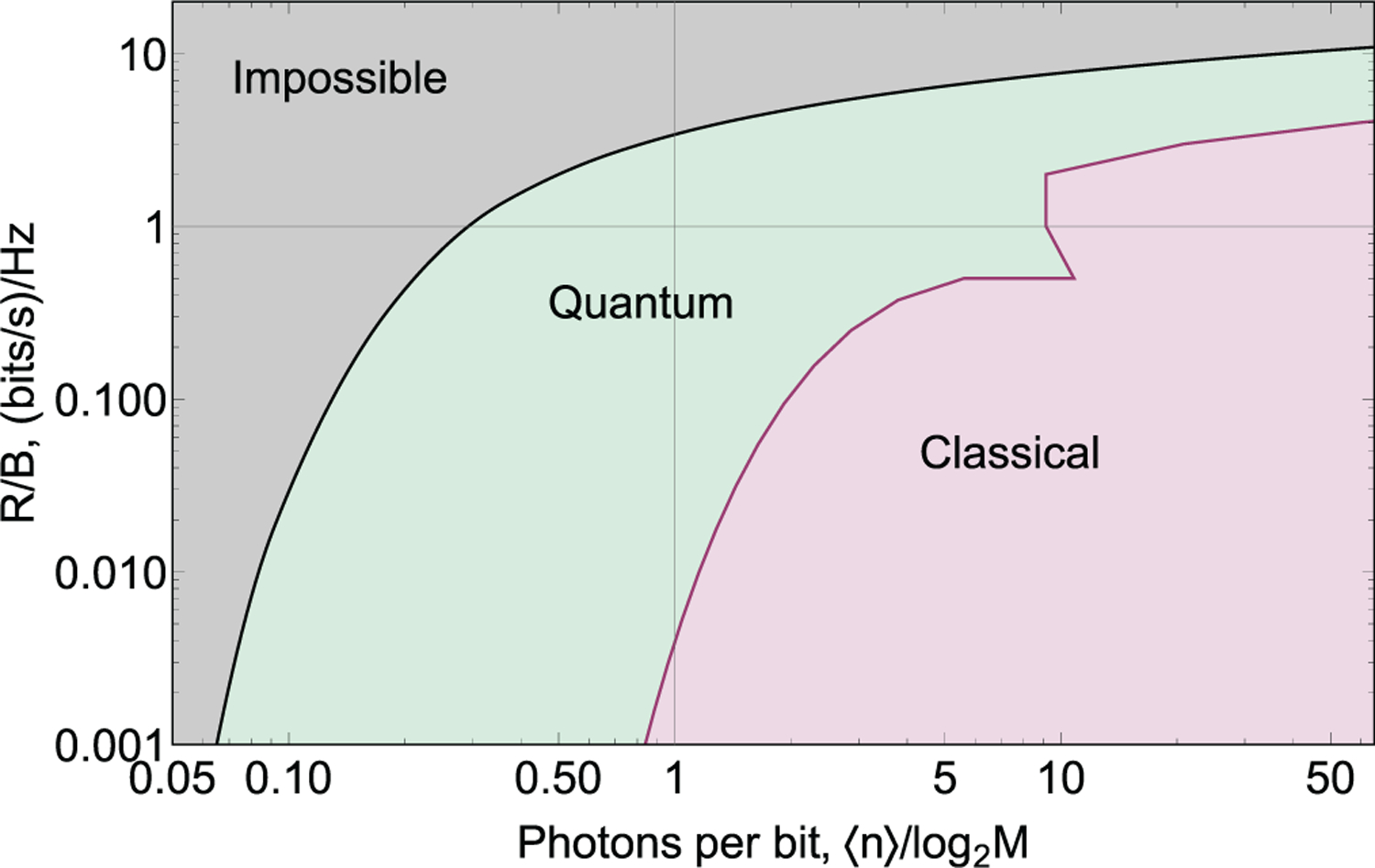
Potential improvement in resource use of quantum-enabled communication over classical technology. The classical resource use is comprised of shot-noise limits (at *P*_*e*_ = 10^−5^) for *M*-ary PSK (values above *R*/*W* = 1) and *M*-ary PPM (values below *R*/*W* = 1), red curve. The potential, but optimistic, quantum bound is Gordon capacity, black curve.

Figure 15 shows the channel resource use required for nearly fault-free communication (*P*_*e*_ = 10^−5^) using traditional modulation methods with ideal classical detection. This theoretical plot does not consider channel noise in a practical communication link, which would make energy requirements significantly greater. The sources of such noise include in-line optical amplifiers, cross-talk between wavelength-multiplexed channels, nonlinear effects in fiber, and dark noise of detectors. On the other hand, this plot does not take error correction into account, which can somewhat relax the energy requirements. Yet, we believe that this curve is a good estimation for the threshold of classical technologies. Some classical systems that are currently near this threshold use single-photon detectors^96–99^ because of their low dark noise.^27^ We see that quantum measurement could potentially reduce channel energy requirements from this threshold by more than one order of magnitude while not requiring more bandwidth.

In certain cases, for instance, for photon-starved communication links, reducing channel energy requirements may be the goal, which is achievable by switching to a quantum measurement at the receiver. However, reducing channel energy requirements does not automatically reduce the total energy consumption of the entire communication link. In fact, the total energy requirements of the state-of-the-art communication link using quantum receivers can be higher than that using classical receivers. Below we discuss if reducing the total energy consumption of communication systems using quantum measurement is fundamentally possible. We also list major technological obstacles that prevent such an energy reduction.

In order to tame the power needs of the telecom links, all components of a communication system should be taken into account. Power requirements of some electronic components scale proportionally to optical power used and those components dominate the power budget of fast (>10 GB/s) optical communication systems.^100^ Quantum measurement reduces the energy of light required to transmit one bit; thus, the power required for those electronic components excluding the receiver reduces proportionally. Displacement quantum receivers require significantly stronger LO than that for the ideal classical homodyne or heterodyne measurement. On the other hand, consider a long-distance fiber link where the optical loss is significant. The energy savings at the transmitter scale proportionally to loss and eventually overcome the additional optical power needs at the receiver. Certain single-photon detectors, such as SPADs, are less energyefficient than classical detectors, yet another issue with quantum receivers. A new generation of single-photon detectors particularly superconductor nanowire detectors can use significantly lower currents to reliably register photons than amplified classical detectors, ultimately dissipating approximately 5 aJ per photon detection.^101,102^ Therefore, on balance, long-distance communication systems can fundamentally be more energy efficient than classical systems. Significant energy savings could also come from a conceptual rethinking of the network topology. Currently, a series of optical amplification stations mitigate light loss in fiber. Amplification stations are used because they require less wall power to operate than a transceiver. If transceivers power requirements could be dropped below that of an amplifier, the topology of the network would significantly change. Given that a large fraction of the optical noise in current networks is due to amplification and optical power-dependent effects (Raman cross-talk, cross- and self- phase modulation, etc.), the quantum-measurement-based communication system can be made nearly noiseless by reducing optical power. Such a nearly noiseless communication system can naturally support the coexistence of classical and quantum communication channels (such as quantum key distribution and entanglement distribution channels). This optimistic outlook faces serious technological challenges. Currently, even the best single-photon detectors at telecom can count fewer than 100 × 10^6^ photons per second. In addition, adaptive algorithms employed in receivers may require extra time to execute. Thus, per-channel data rates may be slower than that of conventional receivers. Wavelength division multiplexing can alleviate this issue, but it will require denser channel “packing” than is currently used. Such packing would require better frequency stabilization of telecom light sources, multiplexers/demultiplexers with better resolution, etc. Some single-photon detectors, such as superconducting nanowire detectors, require a low ambient temperature to operate. Because these detectors generate very little heat when operating, hundreds of such detectors could share the same cooling module.^102^ Also, the efficiency of the state-of-the-art cooling systems is far from theoretically optimal, leaving a lot of room for improvement. Lastly, although most of the proof of principle experiments currently use one laser source for both signal and local oscillator, local laser sources with long coherence times and the phase control should be used to unveil the potential energy saving. To this end, new phase correction protocols are being actively considered. One such protocol^78^ demonstrates phase estimation based on the output of quantum state discrimination, potentially requiring no exchange of phase information between the transmitter and receiver.

In conclusion, in light of the exponential growth of the Internet traffic and capacity crunch,^1,2^ the research of applied practical quantum measurement for communications is of urgent importance. We are cautiously optimistic that quantum technology will be used—either on a global scale or at least for some niche applications in a near future. We hope that our review helped the curious reader to get acquainted with this exciting field.

## Figures and Tables

**Table I.** Assumptions for the channel capacity and SER bounds derivations.

Metric	Channel assumptions	Measurement assumptions	Encoding assumptions

Gordon capacity (Holevo)	Lossless, noiseless	Photon number resolving	Fock states
Helstrom bound	Noiseless	n/a	Any alphabet
Shot Noise limit	Noiseless	Ideal classical	Any alphabet

**Table II.** Quantum and classical SER bounds of different modulation protocols.

Encoding	Optimal classical Receiver	Shot noise limit *P*_SNL_	Helstrom bound *P*_HB_	Ref.

OOK	Direct detection	$\frac{1}{2}e^{-n}$	$\frac{1}{2}\left(1-\sqrt{1-e^{-n}}\right)$	20
BPSK	Homodyne	$\frac{1}{2}(1-\mathrm{erf}(\sqrt{2n}))$	$\frac{1}{2}\left(1-\sqrt{1-e^{-4n}}\right)$	10,34
*M*-PSK	Homodyne	$1-\frac{1}{\pi }\int_{-\pi /M}^{\pi /M}\int_{0}^{\infty }e^{{\left|re^{i\theta }-\sqrt{n}\right|}^{2}}r\;dr\;d\theta$	$1-{\left(\sum_{q=1}^{M}\sqrt{e^{-n}\sum_{m=1}^{M}e^{{\left(1-q\right)}^{\frac{2\pi im}{M}}+ne^{\frac{2\pi im}{M}}}}\right)}^{2}/M^{2}$	24
*M*-PPM	Direct detection	(*M* − 1) *e^−n^/M*	$(M-1){\left(\sqrt{1+(M-1)e^{-n}}-\sqrt{1-e^{-n}}\right)}^{2}/M^{2}$	50,51
M-CFSK	Homodyne	Numerical	Numerical SRM	48,49

**Table III.** Experimentally attained performance of quantum receivers with different encodings. The best energy sensitivity improvement over classical detection is given by the smallest ratio of experimentally measured SER (*P*_E_) to the classical theoretical SNL adjusted for receiver’s system efficiency *η* (*P*_SNL(*η*)_) and to the ideal SNL (*P*_SNL_). The input energy in average number of photons per bit (photons/bit) when this minimum occurs is given in adjacent columns. The energy required to experimentally achieve 10% SER (*P*_E_ = 0:1) is compared to energy requirements to achieve the same with classical measurements, shown as energy efficiency improvement relative to ideal (〈*n*〉_SNL_/〈*n*〉_E_) and adjusted (〈*n*〉_SNL(*η*)_/〈*n*〉_E_) SNLs.

Encoding protocol	$\frac{P_{\text{E}}}{P_{\text{SNL}(\eta )}}@\frac{{\langle n\rangle }_{\text{E}}}{\log_{2}M}$		*η*	$\frac{P_{\text{E}}}{P_{\text{SNL}}}@\frac{{\langle n\rangle }_{\text{E}}}{\log_{2}M}$		⟨*n*⟩_E_/log_2_*M* @*P*_E_ = 0.1	⟨*n*⟩_SNL_/⟨*n*⟩_E_ @*P*_E_ = 0.1	⟨*n*⟩_SNL(*η*)_/⟨*n*⟩_E_ @*P*_E_ = 0.1	References

	dB	photons/bit		dB	photons/bit	photons/bit			
OOK	−2.2	2	0.35	−0.31	0.29	^a^	^a^	^a^	57
	−0.5	0.2	−0.75	0.2	0.7	^a^	^a^	^a^	20
BPSK	−0.77	0.44	0.55	^b^		0.73	0.56	1.03	54
	−0.42	0.21	0.91	−0.15	0.21	0.41	1	1.1	22
	−6	7	0.72	^b^		0.5	0.82	1.14	60
	−4	2.2	0.58	^b^		0.78	0.53	0.91	55 ^c^
4-PPM	−2.3	1.6	0.4	^b^		^a^	^a^	^a^	51
4-PSK	−0.22	1.51	0.53	^b^		^a^	^a^	^a^	59
	−13	4.5	0.72	−6.7	5.5	1.25	1.07	1.48	67
	−27	10	0.72	−14	10	1.25	1.07	1.48	69
	−6.8	2	0.7	−3.7	2	1.02	1.31	1.87	73
	−8.9	4.7	0.65	−1.7	4.2	1.3	1	1.58	68 ^c^
	−6.3	2	0.75	−3.7	2	1	1.33	1.79	75
4-CFSK	−11	2.7	0.75	−7.1	2.7	0.84	1.49	1.98	49
8-CFSK	−7.1	2	0.75	−3.1	2	1	1.25	1.67	49
16-CFSK	−2.6	1	0.75	−0.30	1	1.28	0.95	1.27	49
8-PSK	−3.8	3.1	0.75	−1.86	3.1	2.42	1.27	1.69	75
16-PSK	−2.7	7.4	0.75	−1.2	6.3	7.75	1.15	1.33	75

a Experimentally measured SER is above *P*_E_ = 0.1.

b Experimental SER does not surpass absolute SNL.

c Mark experiments at the telecom wavelength (1550 nm).

## Data Availability

Data sharing is not applicable to this article as no new data were created or analyzed in this study.

## References

[1] Gold H, see https://www.cnn.com/2020/03/19/tech/netflix-internet-overload-eu/index.html for “Netflix and YouTube are Slowing Down in Europe to Keep the Internet from Breaking, 2020.”

[2] Ellis AD, Suibhne NM, Saad D, and Payne DN, Philos. Trans. R. Soc. A 374, 20150191 (2016). 10.1098/rsta.2015.0191 PMC4733922 26809575

[3] Shannon CE, Bell Syst. Tech. J. 27, 379 (1948).

[4] Landauer R, IBM J. Res. Dev. 5, 183 (1961).

[5] Bérut A , Nature 483, 187 (2012). 22398556 10.1038/nature10872

[6] Zurek WH, Rev. Mod. Phys. 75, 715 (2003).

[7] Gordon JP, Proc. IRE 50, 1898 (1962).

[8] Holevo AS, Probl. Peredachi Inf. 15, 3 (1979).

[9] Yuen H, Kennedy R, and Lax M, IEEE Trans. Inf. Theory 21, 125 (1975).

[10] Helstrom CW, J. Stat. Phys. 1, 231 (1969).

[11] Caves CM and Drummond PD, Rev. Mod. Phys. 66, 481 (1994).

[12] Shapiro J, IEEE J. Quantum Electron. 21, 237 (1985).

[13] Shapiro JH, IEEE J. Sel. Top. Quantum Electron. 15, 1547 (2009).

[14] Kikuchi K, J. Lightwave Technol 34, 157 (2016).

[15] Banaszek K, Kunz L, Jachura M, and Jarzyna M, J. Lightwave Technol. 38, 2741 (2020).

[16] Mandel L and Wolf E, Optical Coherence and Quantum Optics (Cambridge University, Cambridge, 1995).

[17] Kennedy RS, “A near-optimum receiver for the binary coherent state quantum channel,” Q. Prog. Rep. 108, 219 (1972).

[18] Dolinar SJ, “An optimum receiver for the binary coherent state quantum channel,” Q. Prog. Rep. 11, 115 (1973).

[19] Bondurant RS, “Near-quantum optimum receivers for the phase-quadrature coherent-state channel,” Opt. Lett. 18, 1896 (1993). 19829439 10.1364/ol.18.001896

[20] Tsujino K , Opt. Express 18, 8107 (2010). 20588655 10.1364/OE.18.008107

[21] Guha S, Habif JL, and Takeoka M, J. Mod. Opt. 58, 257 (2011).

[22] Tsujino K , Phys. Rev. Lett. 106, 250503 (2011). 21770619 10.1103/PhysRevLett.106.250503

[23] Kato K, Osaki M, Sasaki M, and Hirota O, IEEE Trans. Commun. 47, 248 (1999).

[24] Becerra FE , Phys. Rev. A 84, 062324 (2011).

[25] Zuo Y, Li K, and Zhu B, MATEC Web Conf. 61, 06008 (2016).

[26] Müller CR and Marquardt C, New J. Phys. 17, 032003 (2015).

[27] Eisaman MD, Fan J, Migdall A, and Polyakov SV, Rev. Sci. Instrum. 82, 071101 (2011). 21806165 10.1063/1.3610677

[28] Chefles A, Contemp. Phys. 41, 401 (2000).

[29] Bergou J, Herzog U, and Hillery M, “Discrimination of quantum states,” in Quantum State Estimation, Lecture Notes in Physics Vol. 649, edited by Paris M and Řeháček J (Springer Berlin Heidelberg, 2004).

[30] Bergou JA, J. Phys.: Conf. Ser. 84, 012001 (2007).

[31] Barnett SM and Croke S, Adv. Opt. Photonics 1, 238 (2009).

[32] Bergou JA, J. Mod. Opt. 57, 160 (2010).

[33] Bae J and Kwek L-C, J. Phys. A 48, 083001 (2015).

[34] Proakis JG and Salehi M, Digital Communications, 5th ed. (McGraw-Hill, Boston, 2008).

[35] Proakis J and Salehi M, Communication Systems Engineering (Pearson Education, Prentice-Hall, 2002).

[36] Schumacher B and Westmoreland MD, Phys. Rev A 56, 131 (1997). 10.1103/physreva.48.977 9909696

[37] Giovannetti V , Phys. Rev. Lett. 92, 027902 (2004). 14753969 10.1103/PhysRevLett.92.027902

[38] Chung HW, Guha S, and Zheng L, Phys. Rev A 96, 012320 (2017).

[39] Belavkin VP, Radiotekh. Elektron. 20, 1177 (1975).

[40] Hausladen P, Jozsa R, Schumacher B, Westmoreland M, and Wootters WK, Phys. Rev A 54, 1869 (1996). 10.1103/physreva.54.1869 9913673

[41] Xu F , Nat. Commun. 6, 8735 (2015). 26515586 10.1038/ncomms9735 PMC4640067

[42] Caves CM, Phys. Rev. Lett. 45, 75 (1980).

[43] Jaekel MT and Reynaud S, Europhys. Lett. 13, 301 (1990).

[44] Lapidoth A and Moser SM, in Proceedings of the 41st Allerton Conference on Communication, Control and Computing (University of Illinois at Urbana-Champaign, 2003).

[45] Lapidoth A and Moser SM, IEEE Trans. Inf. Theory 55, 303 (2009).

[46] Lapidoth A, Wang L, Shapiro JH, and Venkatesan V, in 25th IEEE Convection of Electrical and Electronics Engineers in Israel (IEEE, 2008), pp. 654–658.

[47] Butman S, Katz J, and Lesh J, IEEE Trans. Commun. 30, 1262 (1982).

[48] Burenkov IA, Tikhonova OV, and Polyakov SV, Optica 5, 227 (2018).

[49] Burenkov I, Jabir M, Battou A, and Polyakov S, PRX Quantum 1, 010308 (2020).

[50] Dolinar SJ, Telecommunications and Data Acquisition Progress Report No. 42–72 (1982).

[51] Chen J, Habif JL, Dutton Z, Lazarus R, and Guha S, Nat. Photonics 6, 374 (2012).

[52] Peres A, Found. Phys. 20, 1441 (1990).

[53] Leibfried D , Science 304, 1476 (2004). 15178794 10.1126/science.1097576

[54] Wittmann C , Phys. Rev. Lett. 101, 210501 (2008). 19113397 10.1103/PhysRevLett.101.210501

[55] Shcherbatenko ML, Elezov MS, Goltsman GN, and Sych DV, Phys. Rev A 101, 032306 (2020).

[56] Geremia J, Phys. Rev A 70, 062303 (2004).

[57] Cook RL, Martin PJ, and Geremia JM, Nature 446, 774 (2007). 17429395 10.1038/nature05655

[58] Takeoka M and Sasaki M, Phys. Rev A 78, 022320 (2008).

[59] Müller CR , New J. Phys. 14, 083009 (2012).

[60] DiMario MT and Becerra FE, Phys. Rev. Lett. 121, 023603 (2018). 30085718 10.1103/PhysRevLett.121.023603

[61] Polyakov SV and Migdall AL, Opt. Express 15, 1390 (2007). 19532370 10.1364/oe.15.001390

[62] Wayne MA, Bienfang JC, and Polyakov SV, Opt. Express 25, 20352 (2017). 29041717 10.1364/OE.25.020352

[63] Takeoka M, Sasaki M, van Loock P, and Lütkenhaus N, Phys. Rev A 71, 022318 (2005).

[64] Sych D and Leuchs G, Phys. Rev. Lett. 117, 200501 (2016). 27886468 10.1103/PhysRevLett.117.200501

[65] Izumi S , Phys. Rev A 86, 042328 (2012).

[66] Izumi S, Takeoka M, Ema K, and Sasaki M, Phys. Rev A 87, 042328 (2013).

[67] Becerra F , Nat. Photonics 7, 147 (2013).

[68] Izumi S, Neergaard-Nielsen JS, Miki S, Terai H, and Andersen UL, Phys. Rev. Appl 13, 054015 (2020).

[69] Becerra F, Fan J, and Migdall A, Nat. Photonics 9, 48 (2015).

[70] Wittmann C, Andersen UL, and Leuchs G, J. Mod. Opt. 57, 213 (2010).

[71] Wittmann C, Andersen UL, Takeoka M, Sych D, and Leuchs G, Phys. Rev. Lett. 104, 100505 (2010). 20366409 10.1103/PhysRevLett.104.100505

[72] Li K, Zuo Y, and Zhu B, IEEE Photonics Technol. Lett. 25, 2182 (2013). 10.1109/LPT.2013.2253765 PMC3922043 24532961

[73] Ferdinand AR, DiMario MT, and Becerra FE, npj Quantum Inf 3, 43 (2017).

[74] Jabir MV, Burenkov IA, Annafianto NFR, Battou A, and Polyakov SV, OSA Continuum 3, 3324 (2020). 10.1364/osac.409200 PMC9074755 35530421

[75] Burenkov IA, Jabir MV, Annafianto NFR, Battou A, and Polyakov SV, in Conference on Lasers and Electro-Optics, FF1D.1 (Optical Society of America, 2020).

[76] DiMario MT, Kunz L, Banaszek K, and Becerra FE, npj Quantum Inf. 5, 65 (2019).

[77] Yuan R, Zhao M, Han S, and Cheng J, IEEE Commun. Lett. 24, 1313 (2020).

[78] DiMario MT and Becerra FE, Phys. Rev. Res. 2, 023384 (2020).

[79] Chesi G, Olivares S, and Paris MGA, Phys. Rev A 97, 032315 (2018).

[80] Izumi S, Neergaard-Nielsen JS, and Andersen UL, J. Phys B 51, 085502 (2018).

[81] Zambra G , Phys. Rev. Lett. 95, 063602 (2005). 16090953 10.1103/PhysRevLett.95.063602

[82] Goldschmidt EA , Phys. Rev A 88, 013822 (2013).

[83] Burenkov IA , Phys. Rev A 95, 053806 (2017).

[84] You C , Appl. Phys. Rev. 7, 021404 (2020).

[85] Habif JL, Jagannathan A, Gartenstein S, Amory P, and Guha S, Opt. Express 29, 7418–7427 (2021). 33726243 10.1364/OE.417989

[86] Izumi S, Neergaard-Nielsen JS, and Andersen UL, Phys. Rev. Lett. 124, 070502 (2020). 32142330 10.1103/PhysRevLett.124.070502

[87] Becerra FE, Fan J, and Migdall A, Nat. Commun. 4, 2028 (2013). 23774177 10.1038/ncomms3028

[88] Izumi S, Neergaard-Nielsen JS, and Andersen UL, arXiv:2009.02558 (2020). 10.1103/PhysRevLett.124.070502 32142330

[89] Izumi S , Sci. Rep. 8, 2999 (2018). 29445101 10.1038/s41598-018-21092-8 PMC5813249

[90] Han R, Bergou JA, and Leuchs G, New J. Phys 20, 043005 (2018).

[91] Namkung M and Kwon Y, Sci. Rep. 9, 19664 (2019). 31873075 10.1038/s41598-019-55589-7 PMC6928166

[92] Han R, Leuchs G, and Bergou JA, Phys. Rev A 101, 032103 (2020).

[93] da Silva MP, Guha S, and Dutton Z, Phys. Rev A 87, 052320 (2013).

[94] Blume-Kohout R, Croke S, and Zwolak M, arXiv:1201.6625 (2012).

[95] Lohani S and Glasser RT, Mach. Learn. 1, 035006 (2020).

[96] Cheng MK, Moision BE, Hamkins J, and Nakashima MA, in IEEE Globecom 2006 (IEEE, 2006), pp. 1–5.

[97] Hopman PI , Proc. SPIE 6304, 120 (2006).

[98] Albota MA, Robinson BS, Caplan DO, Hamilton SA, and Boroson DM, in LEOS 2008–21st Annual Meeting of the IEEE Lasers and Electro-Optics Society (IEEE, 2008), pp. 161–162.

[99] Robinson BS , Opt. Lett. 31, 444 (2006). 16496881 10.1364/ol.31.000444

[100] Kilper D, Guan K, Hinton K, and Ayre R, Proc. IEEE 100, 1168 (2012).

[101] Marsili F , Nat. Photonics 7, 210 (2013).

[102] Shainline JM , J. Appl. Phys. 126, 044902 (2019).

